# Febrile temperature enhances *Plasmodium falciparum* cytoadhesion by disrupting the endothelial glycocalyx

**DOI:** 10.1101/2025.09.07.674757

**Published:** 2025-09-08

**Authors:** Viola Introini, Rory Long, Olawunmi Rashidat Oyerinde, Silvia Sanz Sender, Frank Stein, Gyu Min Hwang, Borja Lopez Gutierrez, Karl Boynton Seydel, Gretchen Birbeck, Maria Bernabeu

**Affiliations:** 1 European Molecular Biology Laboratory (EMBL) Barcelona, Barcelona, Spain; 2 Heidelberg University, Faculty of Biosciences, Heidelberg, Germany; 3 European Molecular Biology Laboratory (EMBL) Heidelberg, Proteomics Core Facility, Heidelberg, Germany.; 4 Heidelberg, University, Faculty of Engineering Sciences, Heidelberg, Germany; 5 Department of Osteopathic Medical Specialties, College of Osteopathic Medicine, Michigan State University, East Lansing; 6 Blantyre Malaria Project, Kamuzu University of Health Sciences, Blantyre, Malawi; 7 Epilepsy Division, Department of Neurology, University of Rochester, Rochester, New York; 8 University Teaching Hospitals Neurology Research Office, Lusaka, Zambia

## Abstract

Fever, a universal host defense in infection and inflammation, paradoxically contributes to neurological complications in malaria. While febrile temperatures enhance the expression of parasite virulence proteins that mediate vascular adhesion and disease severity, its effects in the endothelium remain elusive. Here we present a 3D fever-on-a-chip model that recapitulates human brain and lung microvessels under febrile conditions. Short febrile episodes at 40 °C, common in treated cerebral malaria patients, rapidly enhanced iRBC and immune cell binding under flow. Mechanistically, we demonstrated that this phenotype was driven by endothelial glycocalyx shedding, which exposed endothelial receptors EPCR and ICAM-1. Preserving glycocalyx integrity with a broad MMP inhibitor prevented the temperature-induced rise in cytoadhesion. These findings identify fever as a host-specific amplifier of vascular pathology in malaria and highlight endothelial-protective or antipyretic interventions as important strategies to mitigate febrile microvascular pathology.

Fever is an ancient and metabolically costly host response to infection and inflammation, conserved across vertebrates. This tighly regulated process is initated when injury- and pathogen-associated molecular patterns trigger immune response and cellular secretion of prostaglandin E2 (PGE2)^1,2^, a lipid effector molecule that activates thermoregulatory neuronal circuits in the hypothalamus to rapidly elevate core body temperature^3^. By transiently raising body temperature, fever restricts microbial fitness, slowing replication, and promoting immune clearance of diverse viral and bacterial pathogens^4^. Yet, it is not universally beneficial for the host, and fever prevention improves outcomes in diseases such as sepsis^5^, neurological injury^6^, and malaria^7^.

Fever is a hallmark of malaria infection and a consistent predictor of severity^8,9^. A detrimental role of fever has been recently exemplified by seizure reduction after aggressive antipyretic treatment^10^. Central to severe malaria pathogenesis is the cytoadherence of *P. falciparum*-infected red blood cells (iRBCs) to the vascular endothelium^11^, mediated by clonally variant *P. falciparum* erythrocyte membrane protein 1 (PfEMP1) proteins^12,13^ with binding to intercellular adhesion molecule 1 (ICAM-1) and endothelial protein C receptor (EPCR) strongly linked with severe disease^14^. On the parasite side, febrile temperatures profoundly reshape iRBC biology^15^ inducing cytoskeletal remodelling^16^ and accelerating PfEMP1 surface expression^,17–19^, changes that mechanically translate into rapid increase in iRBC stiffness^20^, adhesion^21,22^, and endothelial contact^19^. At the immune level, fever mobilises host systemic innate and adaptive immune responses, promoting neutrophil and T cell vascular recruitment^23^ and cytokine release^24^. Altogether, these findings position fever as a potent promoter of vascular interaction with both iRBCs and immune cells. Yet studies have focused almost exclusively on pathogen-driven mechanisms, leaving the endothelial response to febrile temperatures poorly understood — a critical gap for host-directed therapeutic strategies.

Here, we employ a bioengineered human fever-on-a-chip microvascular model to dissect how febrile temperatures affect the endothelium and regulate *P. falciparum* iRBC and immune cell adhesion.

## Paediatric patients experience high fever despite antipyretic treatment

**Fig. 1.**
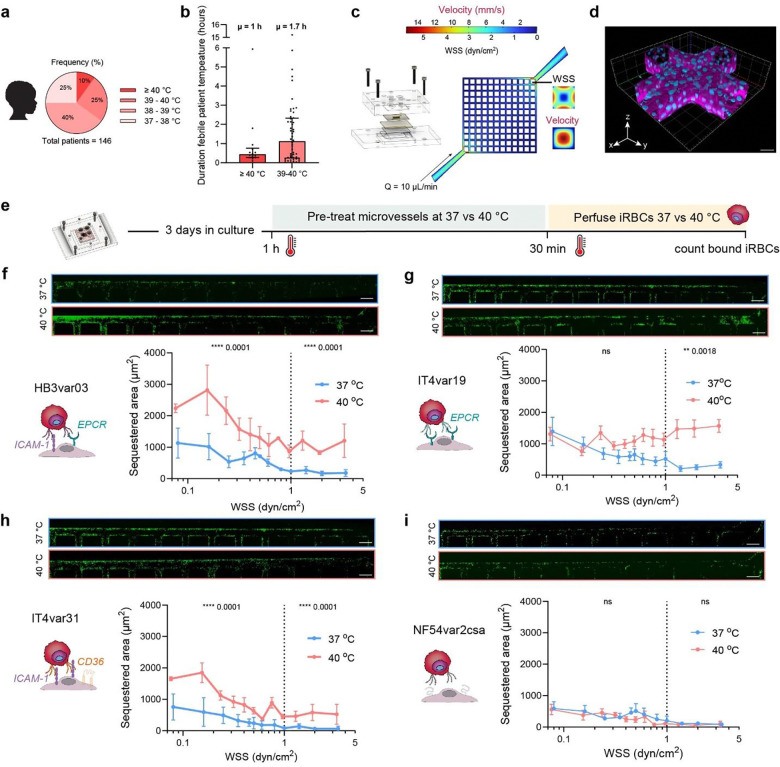
Fever quantification in paediatric malaria patients and *P. falciparum*-iRBC binding at 37 and 40 °C to 3D microvessels. **a,** Distribution of maximum body temperature data during admission from 146 paediatric malaria cases in Zambia and Malawi treated with antipyretics as described in Methods. **b,** Duration of high fever episodes ≥ 40 °C or between 39 and 40 °C. Bar plot represent median and interquartile range; μ indicates group mean. **c,** Simulated mid-plane flow velocity in the grid network prior to collagen remodelling by HBMECs (see Methods). Inset: lumen cross-section velocity and WSS at first branch (black line).**d,** Volumetric reconstruction of a microvessel network grid cross section labelled with VE-cadherin antibody (magenta) and nuclear staining by DAPI (blue). Scale bar = 50 μm. **e,** Schematic representation of the binding assay workflow. **f-i,** Receptor binding schematic and iRBC sequestered areas at 37 °C and 40 °C across WSS for: f, HB3var03; g, IT4var19; h, IT4var31; i, NF54var2csa. Medians are represented by dots and interquartile range by error bars. Statistical analysis of binned regions (< 1 dyn/cm^2^ and ≥ 1 dyn/cm^2^) (dotted line) was determined by Mann-Whitney U test (n = 6 independent biological replicates for HB3var03, IT4var19, and IT4var31; n = 4 for NF54var2csa). Scale bars = 200 μm.

To characterise febrile dynamics in malaria, we analysed continuous temperature recordings from 146 children enrolled in a randomised controlled trial of aggressive antipyretic treatment for malaria Zambia and Malawi. All participants presented central nervous system symptoms including impaired consciousness and/or seizures, and 72–83% fulfilled the clinical definition of cerebral malaria (CM)^7,10^. Core temperature was measured every 2–4 minutes over 72 hours using a monitoring patch. The present analysis was restricted to children in the control group, who received World Health Organisation (WHO)-recommended treatment with acetaminophen for temperatures over 38.5°C. Despite antipyretic therapy, all patients developed fever, with 25% reaching body temperatures between 39–40 °C, and 10% reaching ≥ 40 °C (Fig. 1a). The average fever duration was 1 hour for ≥ 40 °C and 1.7 hours for ≥ 39 °C, with some episodes persisting for several hours (Fig. 1b). These findings demonstrate that short episodes of fever are common in CM patients, even under current WHO-recommended fever management.

## Febrile temperature increases iRBC binding in 3D brain microvessels

To determine whether hyperthermia influences *P. falciparum* binding to endothelial cells, we employed a bioengineered 3D microvascular model that replicates flow-driven cytoadherence. The platform consists of a microfluidic network of 13×13 channels of 120 μm diameter embedded in a collagen hydrogel, recapitulating a wide range of flow velocities spanning 0.4–15 mm s^−1^ – comparable to those found in the brain arteriovenous microcirculation (0.4–8 mm s^−1^)^25,26^ (Fig. 1c). The device displays regions exposed to a wall shear stress (WSS) > 1 dyn/cm^2^ being representative of physiological flow observed in healthy venules (1–5 dyn/cm^2^)^7,17^ and WSS < 1 dyn/cm^2^ being representative of pathological flow conditions likely found in occluded microvasculature^27,28^. Human brain microvascular endothelial cells (HBMECs) are seeded into the network, and after 3 days in culture, they form fully endothelialised 3D microvessels (Fig. 1d). To experimentally model clinical malarial fever episodes, we exposed our engineered 3D human brain microvessel system to 40 °C for 1 hour prior to iRBC perfusion, mimicking the hyperthermic episodes observed in paediatric CM. Since increased temperature affects medium viscosity and density, WSS profiles were simulated at both normothermic (37 °C) and febrile (40 °C) temperatures, showing minimal differences (Extended Data Fig. 1a). After incubation of microvessels for 1 hour at either 37 °C or 40 °C, normothermic *P. falciparum*-iRBC were perfused for 30 minutes at the same temperature as the vessel incubation (Fig. 1e). Binding was quantified along the outer edges of the microvascular grid, and fluorescent areas containing cytoadhered *P. falciparum*-iRBC were defined as the sequestered area (Extended Data Fig. 1b). Multiple clonal lines of *P. falciparum*-iRBCs expressing predominantly single PfEMP1 variants (Extended Data Fig 2a–b), including HB3var03 (EPCR-ICAM-1 binder), IT4var19 (EPCR binder), and IT4var31 (ICAM-1/CD36 binder), exhibited significantly increased binding at 40 °C across multiple WSS conditions (Fig. 1f–h). Temperature did not affect uninfected RBC binding, which remained negligiblele (Extended Data Fig. 3). At febrile temperature, ICAM-1 binders (HB3var03 and IT4var31) presented increased binding across all WSS demonstrated, while the EPCR binder (IT4var19) showed increased binding only at higher WSS (>1 dyn/cm^2^). As a negative control, we used a CSA-binding line expressing var2csa, which mediates placental sequestration. NF54var2csa showed minimal binding at 37 °C and no changes at 40 °C, indicating that increased iRBC binding at fever-range temperatures occurred in parasite lines that presented tropism for the brain endothelium (Fig. 1i). No significant temperature-dependent changes were observed in endothelial junction organisation, microvessel diameter, endothelial cell area, perimeter, or circularity, except for a slight non-signigicant increase in intracellular von Willebrand Factor (vWF) mean fluorescence intensity (MFI), ruling out major heat-induced vessel remodelling (Extended Data Fig. 4a–f). Together, these data show that febrile temperature promotes *P. falciparum*-iRBC binding to endothelial cells in a PfEMP1- and flow-dependent manner.

## EPCR and ICAM-1 mediate temperature-driven iRBC and neutrophil binding without changes in receptor expression

**Fig. 2.**
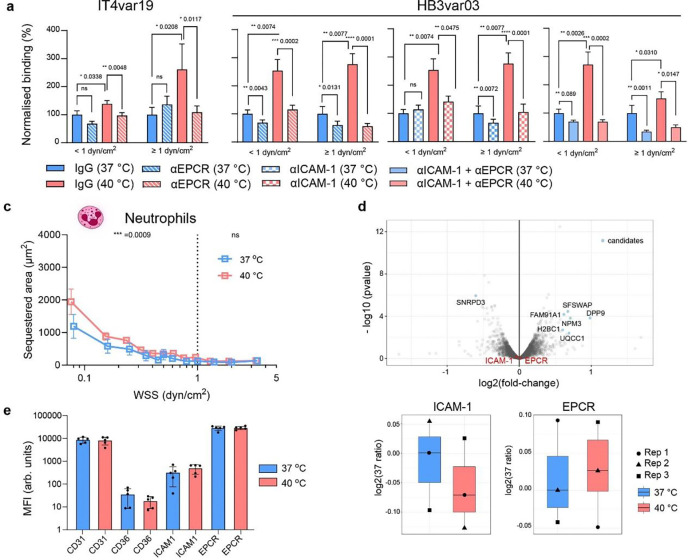
Receptor-mediated binding of *P. falciparum* and neutrophils at febrile temperature occurs without altering endothelial protein level. **a,** Binding of IT4var19 strain iRBCs at 37 °C or 40 °C in the presence of IgG isotype control (filled) or anti-EPCR mAb 252 antibody (stripes). **b**, Binding of HB3var03 strain iRBCs at 37 °C or 40 °C in the presence of IgG isotype control (filled) or both anti-EPCR mAb 252 (stripes), anti-ICAM-1 mAb 15.2 (squares), and both (light colour). Percentage of binding for each treatment is normalised to the respective IgG control at 37 °C. Bars represent mean ± SEM. Statistical analysis for the two WSS regions WSS <1 dyn/cm^2^ and WSS ≥ 1 dyn/cm^2^ using Mann-Whitney U test; n=4–6 independent microvessels. Stratified data can be found in Extended Fig. 5. **c,** Sequestered neutrophil area at 37 °C and 40 °C across WSS (medians as lines and interquartile ranges as error bars). Statistical analysis for the two WSS regions WSS <1 dyn/cm^2^ and WSS ≥ 1 dyn/cm^2^ using Mann-Whitney U test; n = 4 biological replicates per condition. **d,** Volcano plot of differential protein expression of HBMEC monolayer exposed to 37 °C or 40 °C for 1 h (top), with labeled candidates showing statistically significant changes in protein expression. ICAM1 and EPCR differential protein expression (bottom). *n* = 3 independent biological replicates. **e**, MFI of HBMEC surface level expression by flow cytometry after 1h at 37 °C and 40 °C. Bars represent mean ± standard deviation (SD) (n = 5–6 biological replicates).

To investigate whether endothelial receptors are responsible for the mechanism underlying increased sequestration at 40 °C, we inhibited *P. falciparum*-iRBC binding using anti-ICAM-1 and anti-EPCR antibodies. Systematic inhibition of ICAM-1, EPCR, or both at 40 °C significantly reduced binding of both *P. falciparum* lines HB3var03 and IT4var19 compared to an IgG control. Cytoadhesion at 37 and 40 °C did not present any statistical difference after antibody treatment, reaching a baseline residual non-specific binding, specially under pathological low flow (WSS < 1 dyn/cm^2^) (Fig. 2a,b, Extended Data Fig. 5a–d). To examine the broader implications of febrile temperature-induced changes in vascular adhesion, we perfused neutrophils into microvessels pre-incubated at either 37 °C or 40 °C, known to bind to the endothelium in an ICAM-1-mediated manner. Indeed, exposure to 40 °C significantly increased cytoadhesion of neutrophils at pathological low flow but not at higher WSS (Fig. 2c).

To determine whether a temperature-induced increase in expression of ICAM-1 or EPCR contributed to the increase binding of *P. falciparum*-iRBC and neutrophils, we assessed total endothelial proteins by employing quantitative mass spectrometry using tandem mass tag (TMT) labelling. Whole-proteome analysis of HBMEC monolayers exposed to 40 °C for 1 hour revealed minimal changes in protein expression (Figure 2d), with only a handful of significantly upregulated proteins (e.g., SFSWAP, UQCC1, DPP9) and one downregulated transcript (SNRPD3). These proteins are primarily associated with RNA splicing, mitochondrial function, or inflammasome regulation, while protein levels of EPCR and ICAM-1 remained unchanged. Although 1h exposure is probably insufficient for substantial changes at the protein level, further analysis revealed slight increases in heparanase (HSPE), heat shock proteins (HSPs), and prostaglandin synthesis pathway enzimes (PTGs), suggesting early fever-mediated endothelial stress and initiation of heparan sulfate shedding (Extended Data Fig.6). We next used flow cytometry to quantify both the surface levels and the proportion of cells expressing ICAM-1 and EPCR after heat (Figure 2e, Extended Data Fig. 7). No differences were observed for ICAM-1 and EPCR, and CD36, which is minimally expressed in the brain, while CD31 present at 40 °C confirms endothelial cell viability at febrile temperature (Figure 2e, Extended Data Fig. 7). Altogether, *P. falciparum*-iRBC binding at 40 °C is mediated by ICAM-1 and EPCR, however such increase is not explained by differences in the receptor surface expression.

## Febrile temperature promotes endothelial glycocalyx shedding

**Fig. 3.**
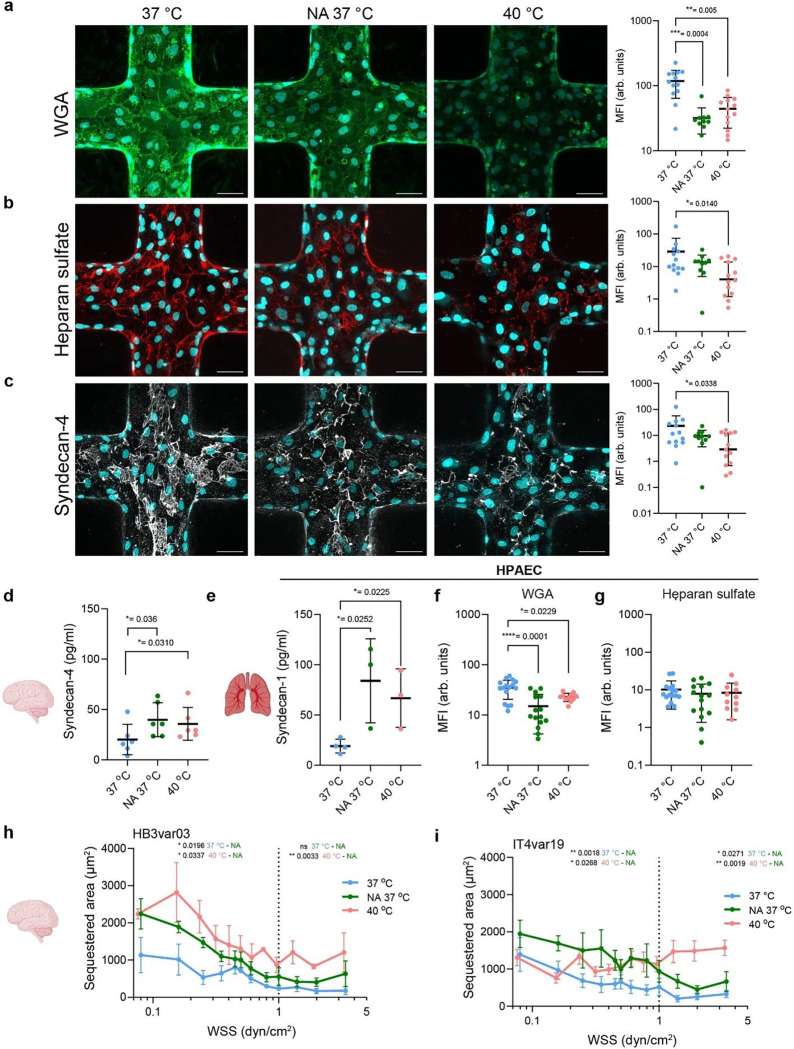
Febrile temperature induces shedding of the endothelial glycocalyx. Immunofluorescence z-stack of microvessel cross sections (left) and MFI normalised by number of cells quantified by DAPI (left) of **a**, sialic acids labelled with FITC-conjugated Wheat Germ Agglutinin (WGA), **b,** heparan sulfate and **c**, syndecan-4; box plots show mean ± SD, n = 4 microvessels per condition, each dots is a region of interest. Scale bars: 50 μm. **d,** Syndecan-4 shedding quantified by ELISA of 3D brain microvessel supernatants following exposure to 37 °C or 40 °C. **e**, Syndecan-1 shedding quantified by ELISA of 3D pulmonary microvessel supernatants. Graphs show mean ± SD, which each dot is from a different microvessel batch. Immunofluorescence MFI quantification of **f**, sialic acids labelled with WGA, and **g**, heparan sulfate in 3D pulmonary microvessels; box plots show mean ± SD, n = 4 microvessels per condition, each dots is a region of interest. Conditions in **a-g** include 37 °C or 40 °C incubation for 1 hour, and NA treatment for 30 min at 37 °C (1 U/mL). Statistical analysis was done by one-way ANOVA with Tukey’s multiple comparisons or pairwise comparisons were performed using the two-sided Mann-Whitney U test. **h-i)** HB3var03 and IT4var19 binding to 3D brain microvessels treated with NA (n = 5 microvessels) compared to 37 °C and 40 °C (n = 6 microvessels). Medians are shown as dots; error bars represent interquartile range. Statistical analysis by Kruskal-Wallis test for binned WSS regions (< 1 dyn/cm^2^ vs ≥ 1 dyn/cm^2^; dotted line).

The glycocalyx is a carbohydrate-rich layer that limits endothelial receptor accessibility^29,30^. To test whether febrile temperature disrupts the endothelial glycocalyx, we exposed HBMECs to 40 °C. Staining revealed a marked loss of sialic acids (Fig. 3a), heparan sulfate (Fig. 3b), and syndecan-4 (Fig. 3c). Syndecan-4, the most abundant proteoglycan, also showed significant shedding (Fig. 3d). The decrease in glycocalyx molecules was comparable to that induced by neuraminidase (NA) treatment, which cleaves terminal sialic acid residues from membrane glycoproteins^31^. To determine if temperature-induced glycocalyx shedding occurs in the vasculature of other organs, we fabricated microvessels from primary human pulmonary artery endothelial cells (HPAECs), an important site of iRBC cytoadhesion^32,33^. Following 40 °C incubation, lung microvessels displayed increased shedding of syndecan-1 (Fig. 3e), a marker of lung vasculature pathology in malaria^34,35^ and other microbial infections^36^. This was accompanied by a decrease in sialic acid but not of heparan sulfate, similar to NA treatment (Fig. 3f, g, Extended Data Fig. 8a, b).

**Fig. 4.**
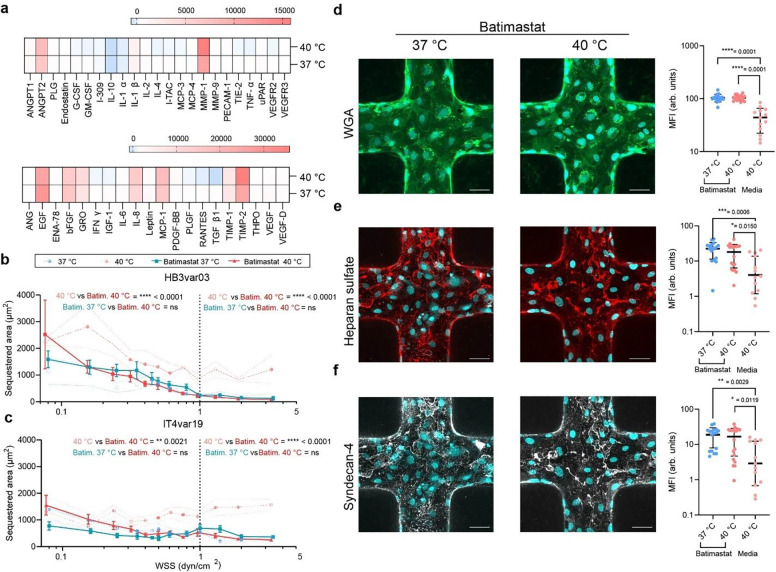
Analysis of MMP1 secretion, *P. falciparum*-iRBC binding and endothelial glycocalyx protection after treatment with the MMP inhibitor, batimastat **a,** Quantification of angiogenesis-related molecule secretion from HBMEC monolayers after 1 h at 37 °C or 40 °C. Supernatants pooled from n=3 biological replicates and normalised as described in Methods. **b-c,** HB3var03 and IT4var19 binding after exposure of batimastat-pre-treated microvessels to 37 °C or 40 °C (n = 4 brain microvessels). Median ± error bars representing interquartile range. Statistical analysis by Kruskal-Wallis test for binned WSS regions (< 1 dyn/cm^2^ vs ≥ 1 dyn/cm^2^; dotted line). **d-f,** Z-projection immunofluorescence images of batimastat pre-treated microvessel junctions exposed to 37 °C or 40 °C and labelled for WGA, heparan sulfate, syndecan-4 and DAPI (cyab) (left) and measured glycocalyx marker MFI normalised by number of cells using DAPI (right) (n = 4 brain microvessels, dots represent quanfied ROI). Statistical analysis by one-way ANOVA with Tukey’s multiple comparisons; box plots show mean ± SD. Scale bars = 50 μm.

To determine whether glycocalyx degradation is responsible for enhanced *P. falciparum*-iRBC binding, we pretreated 3D brain microvessels with NA for 30 minutes at 37 °C, followed by perfusion with HB3var03 and IT4var19 *P. falciparum*-iRBCs. Binding doubled at WSS < 1 dyn/cm^2^ for both lines, reaching levels similar to those observed at 40 °C (Fig. 4h, i). However, NA treatment failed to reproduce increased binding observed at WSS ≥ 1 dyn/cm^2^, specially for the EPCR binder, IT4var19 (Fig. 4i). Incubation with anti-EPCR and ICAM-1 inhibitory antibodies in combination with NA treatment, brought *P. falciparum*-iRBC binding levels back to baseline, thus confirming enhanced PfEMP1-receptor interactions upon glycocalyx disruption (Extended Data Fig. 9a, b). These findings support a model in which breakdown of sialic acids and other glycan structures by fever-induced temperatures act as co-factors that facilitate PfEMP1-mediated adhesion predominantly under low WSS.

## Matrix metalloproteinase inhibition preserves the glycocalyx and reverses temperature-induced cytoadhesion

To investigate the mechanisms driving glycocalyx degradation at febrile temperature, we analysed the secretion of angiogenic mediators, inflammatory cytokines, proteolytic enzymes, and endothelial markers from HBMEC monolayers using a semi-quantitative protein array. After 1 hour at 40 °C, HBMEC exhibited significantly increased secretion of extracellular matrix remodelling proteases, such as matrix metalloproteinase-1 (MMP-1) and vascular inflammatory markers including angiopoietin-2 (ANGPT2). No significant increases in pro-inflammatory cytokines IL-6, IL-1 β, and TNF-α, were observed after 1 hour at 40 °C (Fig. 4a). As MMP-1 has been shown to be linked to glycocalyx degradation^37^, we next examined whether the broad MMP inhibitor batimastat reverses enhanced iRBC cytoadhesion. Indeed, binding of both HB3var03 and IT4var19 *P. falciparum*-iRBCs at 40 °C was significantly reduced by microvessel batimastat pre-treatment at both healthy and pathological WSS, with sequestration levels comparable to those at 37 °C (Fig. 4b, c). Furthermore, we confirmed that batimastat stabilised the glycocalyx, preventing sialic acid, heparan sulfate, and syndecan-4 removal at 40 °C (Fig. 4d–f). Altogether, our results show that short-term exposure of endothelial cells to febrile temperatures promotes iRBC binding by MMP-mediated glycocalyx breakdown, and suggests that protection of the glycocalyx could be a potential therapeutic avenue in the prevention of iRBC cytoadhesion during severe malaria.

## Discussion

Although fever generally exerts protective effects for the host against infection, our findings reveal that during severe malaria infection it may paradoxically amplify pathology by promoting cytoadhesion of *Plasmodium falciparum*–iRBCs. This study shows that brief exposure to high febrile temperatures causes glycocalyx shedding, increasing interactions with blood cells, including *Plasmodium falciparum*-iRBC and neutrophil cytoadhesion. Enhanced cytoadhesion has important clinical implications as elevated sequestered parasite biomass in the microvasculature is a key component of severe malaria^38,39,14^. Increased adhesion during fever could directly worsen disease progression, by promoting vascular obstruction and blood-brain barrier disruption through inflammatory and permeability-inducing pathways^40–43^, as netosis or neutrophil extravasation are linked to CM^44,45^ and acute respiratory distress syndrome^46^, respectively. This aligns with clinical observations linking higher febrile episodes to adverse outcomes, including epilepsy, long-term disabilities and cognitive impairments^47^.

A key finding of this study is that febrile temperature promotes degradation of the endothelial glycocalyx. The glycocalyx is a dynamic network of sugars and proteins lining the vascular endothelium, which forms a barrier that limits direct interaction of endothelial cells with circulating blood cells and vascular components. Its breakdown is common in severe *P. falciparum*^48–51^, *vivax*, and *knowlesi* malaria^35^, with fever emerging as a unifying symptom across malaria infections, despite the three parasites presenting significant differences in biology. Recovery of the glycocalyx is slow, requiring about 20 hours after enzymatic degradation *in vitro*^52^ and up to 7 days *in vivo*^53^. The glycocalyx varies in thickness from 0.2 to 4.5 μm^54^, being more prominent in the brain than in the lung^55,56^. Major cytoadhesion receptors, such as the ~18.7 nm^57,58^ ICAM-1 and the even smaller EPCR, lie well beneath this layer. Our results show that short episodes of fever degrade the brain and lung endothelial glycocalyx, exposing these normally shielded receptors and thereby facilitating *P. falciparum*-iRBC and neutrophil binding under flow^29^. We confirmed this by the enzymatic removal of terminal sialic acids with neuraminidase, which reproduced the febrile binding phenotype under pathological flow conditions (WSS < 1 dyn/cm^2^) for both HB3var03 and IT4var19. Neuraminidase mostly affects the outermost negative-charged glycocalyx layer. By contrast, febrile-range temperatures induced shedding of deeper components, such as syndecan-4 and heparan sulfate. This mechanistic distinction could explain why neuraminidase treatment alone failed to replicate the full febrile binding profile at higher, physiological WSS (≥ 1 dyn/cm^2^), in which *P. falciparum*-iRBCs are only exposed briefly to the endothelial surface.

Our study also shed light on the mechanism of temperature-dependent glycocalyx shedding. Although 1 hour incubation at 40 °C did not elicit a detectable increase in pro-inflammatory cytokines such as IL-6, IL-1 β, IFN γ or TNF-α, or enhanced EPCR and ICAM-1 expression, hyperthermia triggered a rapid release of MMP-1 and Angiopoietin-2, both implicated in glycocalyx breakdown^59^ and CM pathology^60,61,62^. Both molecules are stored in intracellular endothelial vesicles and can rapidly be released^63^, contributing to extracellular matrix remodelling and directly facilitating glycocalyx degradation^64^. Previous findings suggest that temperature-induced MMP1 release could be further amplified by *P. falciparum*, as endothelial cells upregulate MMP1–3 in response to iRBCs at elevated temperatures^65^. Additionally, expression of heparanase, an endoglycosidase that cleaves heparan sulfate and plays a role in neutrophil adhesion during sepsis^55^, was increased following exposure to febrile temperatures. As MMPs can synergise with heparanase to enhance its expression, this may provide an additional mechanism for temperature-dependent glycocalyx breakdown^66^. Importantly, we demonstrated that the MMP inhibitor batimastat can prevent glycocalyx shedding^67^ and the temperature-dependent increase in binding to EPCR and ICAM-1, suggesting potential therapeutic benefit, in agreement with previous mouse experimental cerebral malaria studies showing delayed mortality^68^. However, batimastat’s broad-spectrum activity, lack of specificity and off-target effects limit its clinical development and suggest the use of more specific MMP inhibitors^69^. In the absence of effective host-directed therapies for severe malaria, our findings underscore the need for aggressive antipyretic treatment.

Beyond malaria, hyperthermia profoundly shapes vascular and immune interactions. It accelerates neutrophil migration^70^ and extracellular trap formation^71^, enhances leukocyte adhesion^37,72–75^, and promotes MMP release in sepsis^76^ and stroke^77^, driving basement membrane breakdown. Glycocalyx dysregulation is increasingly recognised as a central determinant of vascular vulnerability, implicated in viral lung injury^30,78^, sepsis^55^, ageing and neurodegeneration^79^. Consistently, hyperthermia worsens outcomes in stroke^9^ and hypoxic–ischemic brain injury^80^ by disrupting the blood–brain barrier and causing brain edema^81^ —paralleling CM pathology. Our findings therefore extend to a broader principle: febrile temperature destabilises the glycocalyx and amplifies microvascular pathology across infectious and inflammatory diseases.

Our flow-based microvessel chip overcomes the restrictions of traditional assays used to study the effects on febrile temperatures on *P. falciparum*-iRBC interactions, that relied on recombinant proteins, which lack glycans or protein ability to freely move and rearrange on the plasma membrane. For example, discrepancies have been found when using force-spectroscopy to measure binding strength to recombinant proteins, showing a weaker binding to recombinant receptors^18,82^ after heating, while binding studies with iRBCs maintained increased cytoadhesion to cell lines^18^. The 3D platform has been previously validated in studies of *P. falciparum* interactions with EPCR and ICAM-1^83^, and in testing inhibitory effect of plasma^84^ or monoclonal antibodies^85^. An advantage of our system, is that it allows to decouple the temperature-effect of fever, independently of elevated pro-inflammatory cytokine levels, key pyrogens that upregulates endothelial receptors, such as ICAM-1. The independent tuning of physical and biological parameters also represents and advantage over hyperthermia animal models. A limitation is that it remains plausable that rapid mechanical modifications at the surface of the *P. falciparum*-iRBC could further contribute to increased cytoadhesion. However, the strong inhibition of binding with batimastat suggests that glycocalyx disruption is a major responsible for increase binding. Future studies should examine the impact of glycocalyx impairment on bond dynamics under flow, the combined effect of hyperthermia and inflammatory signalling in malaria, and extend the model with additional blood–brain barrier cell types or immune cells to directly assess febrile contributions CM pathology.

## Methods

### Body temperature measurements in patients

Body temperature data were collected from 146 children (ages 2–11) who received the WHO recommended treatment with 15 mg/kg acetaminophen every 6 hours as needed for temperatures ≥ 38.5 °C. Participants were enrolled at Queen Elizabeth Central Hospital (Blantyre, Malawi), University Teaching Hospitals Children’s Hospital (Lusaka, Zambia), and Chipata Central Hospital (Chipata, Zambia)^7,10^. All had *P. falciparum* infection confirmed by blood smear or rapid diagnostic test and presented with central nervous system symptoms (impaired consciousness and/or seizures) of whom 72–83% met CM criteria. Temperatures were recorded via a body patch every 2–4 minutes over a 72-hour period, during which most children defervesce. Ethics approvals: Biomedical Research Ethics Committee (Zambia): 003–06-18, University of Rochester Research Subjects Review Board: 00067717, and College of Medicine Research and Ethics Committee (Malawi): P.10/17/2298.

### Plasmodium falciparum culture

*P. falciparum* clones HB3 and IT4 were cultured and selected for expression of PfEMP1 variants HB3VAR03, IT4VAR19, and IT4var31 in human O+ erythrocytes in RPMI-1640 medium (Gibco) containing 25 mM HEPES, 1.5 g/L gentamicin, 0.4 mM hypoxanthine, 26.8 mM sodium bicarbonate, 5 mM glucose, and 10% human type AB+ serum (complete RMPI). Parasites were grown in a gas mixture of 90% N2, 5% CO2, and 5% O2 and parasitemia was regularly checked by Giemsa staining. Cultures were regularly panned and monitored for correct PfEMP1 expression on primary human brain microvascular endothelial cells (HBMECs). *P. falciparum* parasites were synchronized weekly using 5% sorbitol to select for ring-stage parasites.

### 3D brain microvessel fabrication

Primary HBMECs (Cell Systems ACBRI 376) and primary human pulmonary artery endothelial cells (HPAECs) (Lonza CC-2530) were grown in a flask coated with poly-L-lysine (Sigma #P8920) up to passage 9 before they were seeded in microvessels. 3D microvessel devices were prepared as described previously^83^. The top part of the microvessels is generated by injecting type I collagen (7.5 mg/mL) into the space created between the top plexiglass jig and a polydimethylsiloxane (PDMS) mold with a 13-by-13 grid geometry, fabricated using soft lithography. The bottom part consists of a flat layer of collagen, compressed between a flat PDMS stamp and a 22-by-22 mm coverslip positioned on the bottom jig. After 30 minutes of gelation at 37 °C, the PDMS stamps were removed, and the top and bottom jigs were sealed creating a three-dimensional network within the collagen hydrogel. Primary HBMECs or HPAECs were seeded at a concentration of 7×10^6^ cells/mL under gravity-driven flow by adding 8 μL volume increments to the device inlet until reaching full coverage in the microfluidic network. Microvessels were then cultured for up to 3 days and fed every 12 hours by gravity-driven flow before performing *P. falciparum* binding experiments.

### Temperature-dependent parasite binding assay in 3D brain microvessels

*P. falciparum* cultures were enriched for mature-stage iRBCs using a MACS cell separator with LD columns (Miltenyi Biotec 130–042-901) and diluted to 5 × 10^6^/mL in complete RPMI. The iRBCs were labeled with the membrane dye PKH26 Red Fluorescent Cell Linker Midi Kit (Sigma MIDI26–1KT) according to manufacturer’s instructions. For neutrophil binding experiments, neutrophils were isolated from whole blood samples, obtained from the Catalan Blood and Tissue Bank, using the EasySep Direct Human Neutrophil Isolation kit (StemCell 19666) according to manufacturer’s instructions. Isolated neutrophils were labeled with the membrane dye PKH26 and resuspended to a concentration of 2.0×10^6^ neutrophils/mL in RPMI 1640 (Sigma) supplemented with 5% heat-inactivated fetal bovine serum (Gibco), 0.1% vascular endothelial growth factor (Lonza), 0.4% recombinant human FGF (Lonza), 0.1% ascorbic acid (Lonza), 0.1% R3-insulin-like growth factor (Lonza), 0. 1% recombinant human endothelial growth factor (Lonza), 0.5% penicillin/streptomycin (Gibco) and 1% Glutamax (Gibco). The 3D brain microvessels were pre-incubated for 1 hour at either 37 °C or 40 °C. before normothermic iRBCs or neutrophils were perfused for 30 min at 37 °C or 40 °C, respectively, at a flow rate of 10 μl/min using a syringe infusion pump (KD Scientific KDS220). After perfusion, a 5-minute wash with EGM-2MV endothelial medium followed at the same flow rate. Microvessels were fixed in 4% PFA for 20 minutes followed by two 10-minute washes in PBS, and stained with DAPI (8 μg/mL). Each 3D microvessel device was used once for each experimental condition. For iRBC binding inhibition experiments with blocking antibodies, microvessels were pretreated for 20 min at the respective temperature before perfusing iRBCs by gravity-driven flow with mouse anti-human ICAM-1 monoclonal antibody (mAb) 15.2 (Abcam; ab20 [5 μg/ml]), rat anti-human EPCR mAb 252 (Sigma-Aldrich; E6280–200UL [50 μg/ml]), or mouse (Abcam; ab19443) or rat (Thermo Fisher; 14–4301-85) IgG isotype control. For drug inhibition experiments, microvessels were pretreated with the MMP inhibitor batimastat (R&D systems; 2961) at 5μM for 2-h prior to the 1-h incubation at 37 °C or 40 °C.

### Numerical simulation of wall shear stress rates

The flow characteristics of 3D brain microvessels were simulated using COMSOL Multiphysics software, as previously described^83^. Flow in the microvessel network (diameter 120 μm) was assumed to be laminar, and the stationary solver for laminar flow was used with predefined Navier-Stokes equations. Due to the low hematocrit (<0.1%) used during perfusion, flow was assumed to be Newtonian, and wall shear stress (WSS) rates were calculated based on fluid viscosity of water or culture medium at 37 °C (viscosity of 6.922 × 10^−4^ Pa s and density of 993.3 kg/m^3^) and 40 °C (viscosity of 6.539 × 10^−4^ Pa s and density of 992.2 kg/m^3^). The inlet boundary conditions were defined for the perfusion flow rate of 10 μl/min, and the outlet boundary conditions were set at zero pressure.

### Parasite and neutrophil binding quantification

For each device, the edges of the 13-by-13 grid were imaged. A Zeiss LSM 980 AiryScan2 microscope with 10× NA 0.3 objective was used to image cytoadhered iRBCs or neutrophils labeled with PKH26 membrane dye (laser 555 nm) and DAPI-stained parasite and HBMEC nuclei (405 nm laser). DAPI staining was used as control to confirm that HBMEC coverage of the microvessels was uniform. Images were acquired at a 3-μm Z-step size, and projection images of the bottom of the vessel were produced from Z-stacks using Fiji (ImageJ v1.52b) software. The cytoadhered area indicates the area occupied by attached iRBCs across the 12 predefined regions along the edges of the device, as described previously^85^. To minimize flow artifacts, parasite binding was assessed exclusively in the central region of the channel, where flow remains laminar and fully developed, avoiding junctions between branches. Entry and exit regions were excluded, as flow is not fully developed in these regions. Each edge was considered a technical replicate for each device, while each device was considered an independent biological replicate. For statistical analysis, WSS range was divided into 2 regions named pathological^27,28^ (<1 dyn/cm^2^) and physiological (≥1 dyn/cm^2^)^83,86^, in line with flow values observed in postcapillary venules under healthy versus inflammatory conditions.

### Immunofluorescence microscopy of 3D microvessels

Fixed 3D brain microvessels were incubated in Background Buster (Innovex #NB306) for 30 minutes and blocking buffer (2% bovine serum albumin, 0.1% Triton X-100 in PBS) for 1 hour. Blocked microvessels were stained overnight at 4 °C with mouse anti-VE-cadherin primary antibody (Abcam, ab33168) and sheep anti-vWF (Bio-Rad, AHP062) at 1:100 dilution. For Glycocalyx staining, microvessels were blocked with 2% bovine serum albumin in PBS for l hour and stained overnight at 4 °C with 1:50 Human anti-Syndecan-4 antibody (R&D Systems, AF2918-S) and 1:100 anti-Heparan Sulfate (Amsbiom, 370255-S). Microvessels were washed six times for 10 minutes with PBS and incubated for 1 hour at RT with 1:250 Alexa-Fluor 488-, Alexa-Fluor 594- or Alexa-Fluor 647-conjugated secondary antibodies (Invitrogen), containing DAPI 2 μg/mL (ThermoFisher, D21490; 1:250). Following secondary antibody incubation, glycocalyx-stained microvessels were labeled with FITC-conjugated Wheat Germ Agglutinin WGA (VWR 29022–1/ 29022–1; 1:50) for 20 minutes at RT. Microvessels were then washed six times for 10 minutes with PBS and imaging was done using a Zeiss LSM 980 AiryScan 2 confocal microscope with a 20× NA 0.8 objective using a 1-μm Z-step. The 3D rendering of a microvessel cross-section shown in Figure 2 was obtained using Zeiss ZEN v3.3 and Arivis Vision4D v3.5.1 software.

### Human Cytokine Antibody Array

For cytokine analysis, supernatants were collected from 2D HBMEC monolayers from n = 3 biological replicates. A human angiogenesis array (Abcam, ab193655) was performed according to the manufacturer’s instructions. The relative expression of cytokines (measured by chemiluminescence intensity) was compared between samples exposed to 37 °C and 40 °C for 1 hour, corrected to the negative controls on each array, and normalised to the positive controls as suggested by the kit manufacturer. The blots were visualised and imaged (1 min exposure) using the Fusion FX Spectra (Vilber, France).

### ELISA assay

Quantification of Syndecan-4 and Syndecan-1 was performed using the Human Syndecan-4 DuoSet ELISA kit (R&D Systems, DY2918) and Human Syndecan-1 DuoSet ELISA kit (R&D Systems, DY2780), according to manufacturer’s instructions. Media from the outlet reservoir of each device was collected after incubation for 1 hour at 37 °C and 40 °C from N = 3–6 experiments at day 3.

### Proteomics

#### Sample Preparation:

HBMEC cells were cultured on flasks for three to four days until confluent, and then incubated for 1 h either at 37 °C or 40 °C (total of 3 biological replicates per condition). Cells were lysed in cold RIPA buffer with 1× protease inhibitors, incubated on ice for 15 min, sonicated (5 pulses of 5 min at 0/0.5 high, 5°C), and centrifuged at ~14,000 × g for 15 min. Protein concentration was determined using the BCA assay by incubating 20 μL of samples with 200 μL of BCA working reagent (50:1, Reagent A:B) at 37°C for 30 min, followed by absorbance measurement at 562 nm. Samples were subjected to the SP3 protocol^87,88^ on a King Fisher Apex Platform (Thermo Fisher) and peptides were eluted by tryptic digestion (sequencing grade trypsin, Promega) for 5 h at 37°C. Peptides were labelled with TMT6plex^89^ Isobaric Label Reagent (ThermoFisher) according the manufacturer’s instructions. In short, 0.8 mg reagent was dissolved in 42 μL acetonitrile (100%) and 4 μL of stock was added and incubated for 1h room temperature. Followed by quenching the reaction with 5% hydroxylamine for 15 min at RT. Samples were combined and for further sample clean up an OASIS^®^ HLB μElution Plate (Waters) was used. The TMT6-labelled proteome was fractionated by high-pH reversed-phase carried out on an Agilent 1200 Infinity high-performance liquid chromatography system, equipped with a Gemini C18 column (3 μm, 110 Å, 100 × 1.0 mm, Phenomenex). 48 fractions were collected along with the LC separation that were subsequently pooled into 12 fractions. Pooled fractions were dried under vacuum centrifugation and reconstituted in 10 μL 1% formic acid, 4% acetonitrile and then stored at −80 °C until LC-MS analysis.

#### Data acquisition.

Samples were measured on an Q Exactive^™^ Mass Spectrometer (Thermo) coupled to an UltiMate 3000 RSLC nano LC system (Dionex). Sample was concentrated on a C18 μ-Precolumn (Acclaim PepMap 100, 5μm, 300 μm i.d. × 5 mm, 100 Å) and resolved on a nanoEase^™^ M/Z HSS T3 column from Waters (75 μm × 250 mm C18, 1.8 μm, 100 Å). Trapping was carried out at a constant flowrate of 30 μL/min 0.5% trifluoroacetic acid in water for 4 min. Subsequently, peptides were eluted via the analytical column (solvent A: 0.1% formic acid in water, 3% DMSO) with a constant flow of 0.3 μL/min, with increasing percentage of solvent B (0.1% formic acid in acetonitrile, 3% DMSO) from 2% to 8% in 6 min, 8% to 28% in 37 min, from 28% to 40% in 9 min, followed by an increase of B from 40–80% for 3 min and a re-equilibration back to 2% B for 5 min. The peptides were introduced into the mass spectrometer via a Pico-Tip Emitter 360 μm OD × 20 μm ID; 10 μm tip (New Objective) and an applied spray voltage of 2.4 kV. The capillary temperature was at 275°C. Settings for the Q Exactive were: Full mass scan (MS1) was acquired with mass range 375–1200 m/z, profile mode, in the orbitrap, resolution of 70000, fill time 10 ms. AGC target 3E6. Data dependent acquisition (DDA) was performed with the resolution of the Orbitrap set to 17500, fill time 50 ms, AGC target of 9E1 ions. Normalized collision energy of 32, HCD, fixed first mass 110 m/z.

#### Database search.

Fragpipe v21.1 with MSFragger v4.0^90^ was used to process the acquired data, which was searched against the homo sapiens Uniprot proteome database (UP000005640, ID9606, 20594 entries, release October 2022) with common contaminants and reversed sequences included. The following modifications were considered as fixed modification: Carbamidomethyl (C) and TMT6 (K). As variable modifications: Acetyl (Protein N-term), Oxidation (M) and TMT6 (N-term). For the MS1 and MS2 scans a mass error tolerance of 20 ppm was set. Further parameters were: Trypsin as protease with an allowance of maximum two missed cleavages; Minimum peptide length of seven amino acids; The false discovery rate on peptide and protein level was set to 0.01.

#### Data analysis.

The raw output files of FragPipe (protein.tsv files) were processed using the R programming environment (ISBN 3–900051-07–0). Initial data processing included filtering out contaminants and reverse proteins. Only proteins quantified with at least 2 razor peptides (with Razor.Peptides >= 2) were considered for further analysis. 4203 proteins passed the quality control filters. In order to correct for technical variability, batch effects were removed using the ‘removeBatchEffect’ function of the limma package on the log2 transformed raw TMT reporter ion intensities (‘channel’ columns)^91^. Subsequently, normalization was performed using the ‘normalizeVSN’ function of the limma package (VSN - variance stabilization normalization^92^). Differential expression analysis was performed using the moderated t-test provided by the limma package^91^. The model accounted for replicate information by including it as a factor in the design matrix passed to the ‘lmFit’ function. Proteins were annotated as hits if they had a false discovery rate (FDR) below 0.05 and an absolute fold change greater than 2. Proteins were considered candidates if they had an FDR below 0.2 and an absolute fold change greater than 1.5.

### Flow cytometry

HBMECs were cultured in flasks for 3–4 days until confluent, then transferred to 6-well plates and incubated for 1 h at either 37 °C or 40 °C. Cells were rinsed with HBSS and detached using 15 mM EDTA in HBSS. A total of 1 × 10^5^ cells were seeded into wells of a 96-well plate and washed once with PBS containing 0.5% BSA at room temperature. Antibody staining was performed on ice, with each step incubated for 30 minutes. The following antibodies were used: goat anti-human EPCR (5 μg/ml; R&D Systems, AF2245), followed by donkey anti-goat Alexa Fluor 647-conjugated secondary antibody (1:400; Invitrogen, A32849); PE-conjugated anti-human ICAM-1 (2 μl per 10^6^ cells; Miltenyi Biotec, REA266, 130–120-711) and relative isotype control PE-conjugated human IgG1 (Miltenyi, 130–113-471); FITC-conjugated anti-human CD31 (5 μl per 10^5^ cells; BD Pharmingen, 560984) and relative FITC-labelled mouse IgG1 (Abcam, ab18447); FITC-labelled anti-human CD36 (10 μl per 10^5^ cells; Abcam, ab39022) and relative FITC-conjugated mouse IgG1 isotype control (2 μg/ml; Abcam, ab106163). Phosphatidylserine (PS)-exposing apoptotic cells were identified using Annexin V mouse anti-human (10 μg/ml; CoraLite 647, Proteintech, 1E6A8) in Annexin V Binding Buffer. All experiments were performed on live cells, and dead cells were excluded using DAPI (ThermoFisher, D21490; 1:600 dilution). The percentage of positive gated cells and mean fluorescence intensity (MFI) were quantified. Data were analysed using FlowJo v10 software (Tree Star Inc.).

### Statistical analysis

GraphPad Prism (version 10.2.0) was used for statistical analysis. For normally distributed samples, pairwise comparisons were analysed by unpaired t-test with Welch’s correction, while for non-normally distributed samples using Mann-Whitney U test. To compare multiple conditions of normally or non-normally distributed samples we used Kruskal-Wallis test with Dunn’s multiple comparisons test and one-way ANOVA with Tukey’s multiple comparisons, respectively. P values < 0.05 were considered statistically significant. Values are reported as median (Interquartile Interval), mean ± standard deviation or standard deviation of the mean when indicated. Statistical significance is reported as: *p < 0.05, **p < 0.01, ***p < 0.001, ****p < 0.0001. Parasite binding conditions with low variability were tested in four independent 3D microvessel devices, while conditions with high variability were tested in 6 independent devices.

## Supplementary Material

Supplement 1

## Data Availability

The mass spectrometry proteomics data have been deposited to the ProteomeXchange Consortium via the PRIDE [1] partner repository with the dataset identifier PXD063878.

## References

[1] Yamagata K. Coexpression of Microsomal-Type Prostaglandin E Synthase with Cyclooxygenase-2 in Brain Endothelial Cells of Rats during Endotoxin-Induced Fever. J Neurosci 21, 2669–2677 (2001). 11306620 10.1523/JNEUROSCI.21-08-02669.2001 PMC6762538

[2] Eskilsson A. Immune-Induced Fever Is Dependent on Local But Not Generalized Prostaglandin E2 Synthesis in the Brain. J. Neurosci. 37, 5035–5044 (2017). 28438967 10.1523/JNEUROSCI.3846-16.2017 PMC6596481

[3] Lazarus M. EP3 prostaglandin receptors in the median preoptic nucleus are critical for fever responses. Nat Neurosci 10, 1131–1133 (2007). 17676060 10.1038/nn1949

[4] Evans S. S., Repasky E. A. & Fisher D. T. Fever and the thermal regulation of immunity: the immune system feels the heat. Nat Rev Immunol 15, 335–349 (2015). 25976513 10.1038/nri3843 PMC4786079

[5] Launey Y., Nesseler N., Mallédant Y. & Seguin P. Clinical review: fever in septic ICU patients--friend or foe? Crit Care 15, 222 (2011). 21672276 10.1186/cc10097 PMC3218963

[6] Polderman K. H. Induced hypothermia and fever control for prevention and treatment of neurological injuries. Lancet 371, 1955–1969 (2008). 18539227 10.1016/S0140-6736(08)60837-5

[7] Chilombe M. B. Aggressive antipyretics in central nervous system malaria: Study protocol of a randomized-controlled trial assessing antipyretic efficacy and parasite clearance effects (Malaria FEVER study). PLOS ONE 17, e0268414 (2022). 36206262 10.1371/journal.pone.0268414 PMC9543763

[8] Suz P., Vavilala M. S., Souter M., Muangman S. & Lam A. M. Clinical Features of Fever Associated With Poor Outcome in Severe Pediatric Traumatic Brain Injury. Journal of Neurosurgical Anesthesiology 18, 5 (2006). 16369134 10.1097/01.ana.0000189079.26212.37

[9] Greer D. M., Funk S. E., Reaven N. L., Ouzounelli M. & Uman G. C. Impact of Fever on Outcome in Patients With Stroke and Neurologic Injury. Stroke 39, 3029–3035 (2008). 18723420 10.1161/STROKEAHA.108.521583

[10] Birbeck G. L. Acetaminophen and Ibuprofen in Pediatric Central Nervous System Malaria: A Randomized Clinical Trial. JAMA Neurology 81, 857–865 (2024). 38857015 10.1001/jamaneurol.2024.1677 PMC11165415

[11] Miller L. H., Baruch D. I., Marsh K. & Doumbo O. K. The pathogenic basis of malaria. Nature 415, 673–679 (2002). 11832955 10.1038/415673a

[12] Smith J. D. Switches in Expression of Plasmodium falciparum var Genes Correlate with Changes in Antigenic and Cytoadherent Phenotypes of Infected Erythrocytes. Cell 82, 101–110 (1995). 7606775 10.1016/0092-8674(95)90056-x PMC3730239

[13] Su X. Z. The large diverse gene family var encodes proteins involved in cytoadherence and antigenic variation of Plasmodium falciparum-infected erythrocytes. Cell 82, 89–100 (1995). 7606788 10.1016/0092-8674(95)90055-1

[14] Sahu P. K. Determinants of brain swelling in pediatric and adult cerebral malaria. JCI Insight 6, e145823 (2021). 34549725 10.1172/jci.insight.145823 PMC8492338

[15] Jones D. Physiological febrile heat stress increases cytoadhesion through increased protein trafficking of Plasmodium falciparum surface proteins into the red blood cell. eLife 14, (2025). 10.7554/eLife.107860 PMC13171106 42126964

[16] Oakley M. S. M. Molecular Factors and Biochemical Pathways Induced by Febrile Temperature in Intraerythrocytic *Plasmodium falciparum* Parasites. Infect Immun 75, 2012–2025 (2007). 17283083 10.1128/IAI.01236-06 PMC1865691

[17] Udomsangpetch R. Febrile temperatures induce cytoadherence of ring-stage *Plasmodium falciparum* -infected erythrocytes. Proc. Natl. Acad. Sci. U.S.A. 99, 11825–11829 (2002). 12177447 10.1073/pnas.172398999 PMC129353

[18] Carvalho P. A., Diez-Silva M., Chen H., Dao M. & Suresh S. Cytoadherence of erythrocytes invaded by Plasmodium falciparum: Quantitative contact-probing of a human malaria receptor. Acta Biomaterialia 9, 6349–6359 (2013). 23376131 10.1016/j.actbio.2013.01.019 PMC3870279

[19] Zhang R., Chandramohanadas R., Lim C. T. & Dao M. Febrile Temperature Elevates the Expression of Phosphatidylserine on Plasmodium falciparum (FCR3CSA) Infected Red Blood Cell Surface Leading to Increased Cytoadhesion. Sci Rep 8, 15022 (2018). 30302009 10.1038/s41598-018-33358-2 PMC6177484

[20] Marinkovic M. Febrile temperature leads to significant stiffening of *Plasmodium falciparum* parasitized erythrocytes. American Journal of Physiology-Cell Physiology 296, C59–C64 (2009). 18596215 10.1152/ajpcell.00105.2008 PMC2636996

[21] Lubiana P. Adhesion between P. falciparum infected erythrocytes and human endothelial receptors follows alternative binding dynamics under flow and febrile conditions. Sci Rep 10, 4548 (2020). 32161335 10.1038/s41598-020-61388-2 PMC7066226

[22] Dörpinghaus M. Stringent Selection of Knobby Plasmodium falciparum-Infected Erythrocytes during Cytoadhesion at Febrile Temperature. Microorganisms 8, 174 (2020). 31991814 10.3390/microorganisms8020174 PMC7074740

[23] Tulapurkar M. E. Febrile-Range Hyperthermia Modifies Endothelial and Neutrophilic Functions to Promote Extravasation. Am J Respir Cell Mol Biol 46, 807–814 (2012). 22281986 10.1165/rcmb.2011-0378OC PMC3380289

[24] Keitelman I. A. Short-Term Fever-Range Hyperthermia Accelerates NETosis and Reduces Pro-inflammatory Cytokine Secretion by Human Neutrophils. Front. Immunol. 10, (2019). 10.3389/fimmu.2019.02374 PMC6813732 31681277

[25] Koutsiaris A. G. Volume flow and wall shear stress quantification in the human conjunctival capillaries and post-capillary venules in vivo. Biorheology 44, 375–386 (2007). 18401076

[26] Itoh Y. & Suzuki N. Control of brain capillary blood flow. J Cereb Blood Flow Metab 32, 1167–1176 (2012). 22293984 10.1038/jcbfm.2012.5 PMC3390803

[27] Kaul D. K., Roth E. J., Nagel R. L., Howard R. J. & Handunnetti S. M. Rosetting of in *Plasmodium falciparum*-infected red blood cells with uninfected red blood cells enhances microvascular obstruction under flow conditions. Blood 78, 812–819 (1991). 1859893

[28] Sahu P. K. Pathogenesis of cerebral malaria: new diagnostic tools, biomarkers, and therapeutic approaches. Front Cell Infect Microbiol 5, 75 (2015). 26579500 10.3389/fcimb.2015.00075 PMC4621481

[29] Hempel C., Wang C. W., Kurtzhals J. A. & Staalsø T. Binding of Plasmodium falciparum to CD36 can be shielded by the glycocalyx. Malar J 16, 193 (2017). 28486940 10.1186/s12936-017-1844-6 PMC5424350

[30] Targosz-Korecka M. Endothelial glycocalyx shields the interaction of SARS-CoV-2 spike protein with ACE2 receptors. Sci Rep 11, 12157 (2021). 34108510 10.1038/s41598-021-91231-1 PMC8190434

[31] Introini V., Carciati A., Tomaiuolo G., Cicuta P. & Guido S. Endothelial glycocalyx regulates cytoadherence in Plasmodium falciparum malaria. J. R. Soc. Interface 15, 20180773 (2018). 30958233 10.1098/rsif.2018.0773 PMC6303788

[32] Corbett C. E., Duarte M. I., Lancellotti C. L., Silva M. A. & Andrade Júnior H. F. Cytoadherence in human falciparum malaria as a cause of respiratory distress. J Trop Med Hyg 92, 112–120 (1989). 2651687

[33] Van den Steen P. E. Pathogenesis of malaria-associated acute respiratory distress syndrome. Trends in Parasitology 29, 346–358 (2013). 23742967 10.1016/j.pt.2013.04.006

[34] Bush M. A. Degradation of endothelial glycocalyx in Tanzanian children with Falciparum malaria. FASEB J 35, e21805 (2021). 34403544 10.1096/fj.202100277RR PMC8375618

[35] Barber B. E. Endothelial glycocalyx degradation and disease severity in Plasmodium vivax and Plasmodium knowlesi malaria. Sci Rep 11, 9741 (2021). 33963210 10.1038/s41598-021-88962-6 PMC8105350

[36] Park P. W., Pier G. B., Hinkes M. T. & Bernfield M. Exploitation of syndecan-1 shedding by Pseudomonas aeruginosa enhances virulence. Nature 411, 98–102 (2001). 11333985 10.1038/35075100

[37] Mulivor A. W. & Lipowsky H. H. Inhibition of Glycan Shedding and Leukocyte-Endothelial Adhesion in Postcapillary Venules by Suppression of Matrixmetalloprotease Activity with Doxycycline. Microcirculation 16, 657–666 (2009). 19905966 10.3109/10739680903133714

[38] Bernabeu M. Severe adult malaria is associated with specific PfEMP1 adhesion types and high parasite biomass. Proc Natl Acad Sci U S A 113, E3270–3279 (2016). 27185931 10.1073/pnas.1524294113 PMC4988613

[39] Kessler A. Linking EPCR-Binding PfEMP1 to Brain Swelling in Pediatric Cerebral Malaria. Cell Host Microbe 22, 601–614.e5 (2017). 29107642 10.1016/j.chom.2017.09.009 PMC5783720

[40] Gillrie M. R. Src-family kinase–dependent disruption of endothelial barrier function by Plasmodium falciparum merozoite proteins. Blood 110, 3426–3435 (2007). 17693580 10.1182/blood-2007-04-084582 PMC2200906

[41] Gallego-Delgado J. Angiotensin receptors and β-catenin regulate brain endothelial integrity in malaria. J Clin Invest 126, 4016–4029 (2016). 27643439 10.1172/JCI87306 PMC5096829

[42] Howard C., Joof F., Hu R., Smith J. D. & Zheng Y. Probing cerebral malaria inflammation in 3D human brain microvessels. Cell Rep 42, 113253 (2023). 37819760 10.1016/j.celrep.2023.113253 PMC10710687

[43] Piatti L. Plasmodium falciparum egress disrupts endothelial junctions and activates JAK-STAT signaling in a microvascular 3D blood-brain barrier model. Nat Commun 16, 7262 (2025). 40769972 10.1038/s41467-025-62514-2 PMC12328663

[44] Knackstedt S. L. Neutrophil extracellular traps drive inflammatory pathogenesis in malaria. Sci Immunol 4, eaaw0336 (2019). 31628160 10.1126/sciimmunol.aaw0336 PMC6892640

[45] Du X. PRL2 regulates neutrophil extracellular trap formation which contributes to severe malaria and acute lung injury. Nat Commun 15, 881 (2024). 38286811 10.1038/s41467-024-45210-5 PMC10825202

[46] Yang S.-C., Tsai Y.-F., Pan Y.-L. & Hwang T.-L. Understanding the role of neutrophils in acute respiratory distress syndrome. Biomed J 44, 439–446 (2021). 33087299 10.1016/j.bj.2020.09.001 PMC7481802

[47] Birbeck G. L. Blantyre Malaria Project Epilepsy Study (BMPES) of neurological outcomes in retinopathy-positive paediatric cerebral malaria survivors: a prospective cohort study. The Lancet Neurology 9, 1173–1181 (2010). 21056005 10.1016/S1474-4422(10)70270-2 PMC2988225

[48] Yeo T. W. Glycocalyx breakdown is associated with severe disease and fatal outcome in *Plasmodium falciparum* malaria. Clin Infect Dis 69, 1712–1720 (2019). 30753363 10.1093/cid/ciz038 PMC6821254

[49] Yeo T. W. Glycocalyx breakdown is increased in African children with cerebral and uncomplicated falciparum malaria. The FASEB Journal 33, 14185–14193 (2019). 31658834 10.1096/fj.201901048RR PMC6894053

[50] Georgiadou A. & Cunnington A. J. Shedding of the vascular endothelial glycocalyx: a common pathway to severe malaria? Clin Infect Dis 69, 1721–1723 (2019). 30698670 10.1093/cid/ciz043

[51] Lyimo E. In Vivo Imaging of the Buccal Mucosa Shows Loss of the Endothelial Glycocalyx and Perivascular Hemorrhages in Pediatric Plasmodium falciparum Malaria. Infect Immun 88, e00679–19 (2020). 31871101 10.1128/IAI.00679-19 PMC7035942

[52] Giantsos-Adams K. M. Heparan Sulfate Regrowth Profiles Under Laminar Shear Flow Following Enzymatic Degradation. Cell Mol Bioeng 6, 160–174 (2013). 23805169 10.1007/s12195-013-0273-z PMC3689914

[53] Potter D. R., Jiang J. & Damiano E. R. The recovery time course of the endothelial-cell glycocalyx in vivo and its implications in vitro. Circ Res 104, 1318–1325 (2009). 19443840 10.1161/CIRCRESAHA.108.191585 PMC2764238

[54] Reitsma S., Slaaf D. W., Vink H., van Zandvoort M. A. M. J. & oude Egbrink M. G. A. The endothelial glycocalyx: composition, functions, and visualization. Pflugers Arch 454, 345–359 (2007). 17256154 10.1007/s00424-007-0212-8 PMC1915585

[55] Schmidt E. P. The pulmonary endothelial glycocalyx regulates neutrophil adhesion and lung injury during experimental sepsis. Nat Med 18, 1217–1223 (2012). 22820644 10.1038/nm.2843 PMC3723751

[56] Ando Y. Brain-Specific Ultrastructure of Capillary Endothelial Glycocalyx and Its Possible Contribution for Blood Brain Barrier. Sci Rep 8, 17523 (2018). 30504908 10.1038/s41598-018-35976-2 PMC6269538

[57] Kirchhausen T., Staunton D. E. & Springert T. A. Location of the domains of ICAM-1 by immunolabeling and single-molecule electron microscopy. Journal of Leukocyte Biology 53, 342–346 (1993). 8095966 10.1002/jlb.53.3.342

[58] Jun C.-D. Ultrastructure and Function of Dimeric, Soluble Intercellular Adhesion Molecule-1 (ICAM-1)*. Journal of Biological Chemistry 276, 29019–29027 (2001). 11390397 10.1074/jbc.M103394200

[59] Lukasz A. Endothelial glycocalyx breakdown is mediated by angiopoietin-2. Cardiovascular Research 113, 671–680 (2017). 28453727 10.1093/cvr/cvx023

[60] Deininger M. H., Winkler S., Kremsner P. G., Meyermann R. & Schluesener H. J. Angiogenic proteins in brains of patients who died with cerebral malaria. Journal of Neuroimmunology 142, 101–111 (2003). 14512169 10.1016/s0165-5728(03)00250-9

[61] Jain V. Plasma levels of angiopoietin-1 and −2 predict cerebral malaria outcome in Central India. Malaria Journal 10, 383 (2011). 22192385 10.1186/1475-2875-10-383 PMC3286486

[62] Conroy A. L. Angiopoietin-2 levels are associated with retinopathy and predict mortality in Malawian children with cerebral malaria: A retrospective case–control study*. Critical Care Medicine 40, 952 (2012). 22343839 10.1097/CCM.0b013e3182373157 PMC3284252

[63] Taraboletti G. Shedding of the Matrix Metalloproteinases MMP-2, MMP-9, and MT1-MMP as Membrane Vesicle-Associated Components by Endothelial Cells. The American Journal of Pathology 160, 673–680 (2002). 11839588 10.1016/S0002-9440(10)64887-0 PMC1850663

[64] Endo K. Cleavage of syndecan-1 by membrane type matrix metalloproteinase-1 stimulates cell migration. J Biol Chem 278, 40764–40770 (2003). 12904296 10.1074/jbc.M306736200

[65] Allweier J. Cytoadhesion of Plasmodium falciparum-Infected Red Blood Cells Changes the Expression of Cytokine-, Histone- and Antiviral Protein-Encoding Genes in Brain Endothelial Cells. Mol Microbiol 122, 948–967 (2024). 39630601 10.1111/mmi.15331 PMC11658792

[66] Fitzgerald M., Hayward I. P., Thomas A. C., Campbell G. R. & Campbell J. H. Matrix metalloproteinase can facilitate the heparanase-induced promotion of phenotype change in vascular smooth muscle cells. Atherosclerosis 145, 97–106 (1999). 10428300 10.1016/s0021-9150(99)00019-2

[67] Ramnath R. D. Blocking matrix metalloproteinase-mediated syndecan-4 shedding restores the endothelial glycocalyx and glomerular filtration barrier function in early diabetic kidney disease. Kidney Int 97, 951–965 (2020). 32037077 10.1016/j.kint.2019.09.035 PMC7184681

[68] Hempel C., Sporring J. & Kurtzhals J. A. L. Experimental cerebral malaria is associated with profound loss of both glycan and protein components of the endothelial glycocalyx. The FASEB Journal 33, 2058–2071 (2019). 30226810 10.1096/fj.201800657R

[69] Dove A. MMP inhibitors: Glimmers of hope amidst clinical failures. Nature Medicine 8, 95–95 (2002). 10.1038/nm0202-95 11821877

[70] Khachaturyan G. Temperature-sensitive migration dynamics in neutrophil-differentiated HL-60 cells. Sci Rep 12, 7053 (2022). 35488042 10.1038/s41598-022-10858-w PMC9054779

[71] Janko J. Neutrophil extracellular traps formation and clearance is enhanced in fever and attenuated in hypothermia. Front. Immunol. 14, (2023). 10.3389/fimmu.2023.1257422 PMC10577177 37849757

[72] Lefor A. T. Hyperthermia increases intercellular adhesion molecule-1 expression and lymphocyte adhesion to endothelial cells. Surgery 116, 214–220; discussion 220–221 (1994). 7914035

[73] Chen Q. Thermal Facilitation of Lymphocyte Trafficking Involves Temporal Induction of Intravascular ICAM-1. Microcirculation 16, 143–158 (2009). 19031292 10.1080/10739680802353850 PMC4728711

[74] Chen Q. Fever-range thermal stress promotes lymphocyte trafficking across high endothelial venules via an interleukin 6 trans-signaling mechanism. Nat Immunol 7, 1299–1308 (2006). 17086187 10.1038/ni1406

[75] Shah A. Cytokine and adhesion molecule expression in primary human endothelial cells stimulated with fever-range hyperthermia. Int J Hyperthermia 18, 534–551 (2002). 12537753 10.1080/02656730210157843

[76] Yazdan-Ashoori P. Elevated plasma matrix metalloproteinases and their tissue inhibitors in patients with severe sepsis. J Crit Care 26, 556–565 (2011). 21439766 10.1016/j.jcrc.2011.01.008

[77] Alam M., Mohammad A., Rahman S., Todd K. & Shuaib A. Hyperthermia up-regulates matrix metalloproteinases and accelerates basement membrane degradation in experimental stroke. Neuroscience Letters 495, 135–139 (2011). 21443925 10.1016/j.neulet.2011.03.056

[78] Taghavi S. Glycocalyx degradation and the endotheliopathy of viral infection. PLOS ONE 17, e0276232 (2022). 36260622 10.1371/journal.pone.0276232 PMC9581367

[79] Shi S. M. Glycocalyx dysregulation impairs blood–brain barrier in ageing and disease. Nature 639, 985–994 (2025). 40011765 10.1038/s41586-025-08589-9 PMC11946907

[80] Laptook A. R. & Corbett R. J. T. The effects of temperature on hypoxic-ischemic brain injury. Clinics in Perinatology 29, 623–649 (2002). 12516739 10.1016/s0095-5108(02)00057-x

[81] Kiyatkin E. A. & Sharma H. S. Permeability of the blood–brain barrier depends on brain temperature. Neuroscience 161, 926–939 (2009). 19362131 10.1016/j.neuroscience.2009.04.004 PMC2694729

[82] Lim Y. B. Temperature-Induced Catch-Slip to Slip Bond Transit in *Plasmodium falciparum*-Infected Erythrocytes. Biophysical Journal 118, 105–116 (2020). 31813540 10.1016/j.bpj.2019.11.016 PMC6950811

[83] Bernabeu M. Binding Heterogeneity of Plasmodium falciparum to Engineered 3D Brain Microvessels Is Mediated by EPCR and ICAM-1. mBio 10, e00420–19 (2019). 31138740 10.1128/mBio.00420-19 PMC6538777

[84] Joof F. Plasma From Older Children in Malawi Inhibits Plasmodium falciparum Binding in 3-Dimensional Brain Microvessels. J Infect Dis 230, e1402–e1411 (2024). 38875153 10.1093/infdis/jiae315 PMC11646604

[85] Reyes R. A. Broadly inhibitory antibodies to severe malaria virulence proteins. Nature 636, 182–189 (2024). 39567685 10.1038/s41586-024-08220-3 PMC12338075

[86] Lipowsky H. H. Microvascular Rheology and Hemodynamics. Microcirculation 12, 5–15 (2005). 15804970 10.1080/10739680590894966

[87] Hughes C. S. Ultrasensitive proteome analysis using paramagnetic bead technology. Molecular Systems Biology 10, 757 (2014). 25358341 10.15252/msb.20145625 PMC4299378

[88] Hughes C. S. Single-pot, solid-phase-enhanced sample preparation for proteomics experiments. Nat Protoc 14, 68–85 (2019). 30464214 10.1038/s41596-018-0082-x

[89] Dayon L. Relative Quantification of Proteins in Human Cerebrospinal Fluids by MS/MS Using 6-Plex Isobaric Tags. Anal. Chem. 80, 2921–2931 (2008). 18312001 10.1021/ac702422x

[90] Kong A. T., Leprevost F. V., Avtonomov D. M., Mellacheruvu D. & Nesvizhskii A. I. MSFragger: ultrafast and comprehensive peptide identification in mass spectrometry–based proteomics. Nat Methods 14, 513–520 (2017). 28394336 10.1038/nmeth.4256 PMC5409104

[91] Ritchie M. E. limma powers differential expression analyses for RNA-sequencing and microarray studies. Nucleic Acids Research 43, e47 (2015). 25605792 10.1093/nar/gkv007 PMC4402510

[92] Huber W., von Heydebreck A., Sültmann H., Poustka A. & Vingron M. Variance stabilization applied to microarray data calibration and to the quantification of differential expression. Bioinformatics 18 Suppl 1, S96–104 (2002). 12169536 10.1093/bioinformatics/18.suppl_1.s96

