# Supplementary material for: Febrile temperature enhances Plasmodium falciparum cytoadhesion by disrupting the endothelial glycocalyx
Source: bioRxiv. 2025 Sep 8:2025.09.07.674757. Preprint. [Version 1] doi: 10.1101/2025.09.07.674757 (PMC12440002; doi:10.1101/2025.09.07.674757)
Supplement: Supplement 1 [file media-1.pdf]

Febrile temperature promotes *Plasmodium falciparum* vascular cytoadhesion by disrupting the endothelial glycocalyx

Viola Introini[1 *], Rory Long[1,2], Olawunmi Rashidat Oyerinde[1], Silvia Sanz Sender[1], Frank Stein[3], Gyu Min Hwang[1,4], Borja Lopez Gutierrez[1], Karl Boynton Seydel[5,6,], Gretchen Birbeck[6,7,8], Maria Bernabeu[1*]

[1] European Molecular Biology Laboratory (EMBL) Barcelona, Barcelona, Spain

[2] Heidelberg University, Faculty of Biosciences, Heidelberg, Germany

[3] European Molecular Biology Laboratory (EMBL) Heidelberg, Proteomics Core Facility, Heidelberg, Germany.

[4] Heidelberg, University, Faculty of Engineering Sciences, Heidelberg, Germany

[5] Department of Osteopathic Medical Specialties, College of Osteopathic Medicine, Michigan State University, East Lansing

[6] Blantyre Malaria Project, Kamuzu University of Health Sciences, Blantyre, Malawi

[7] Epilepsy Division, Department of Neurology, University of Rochester, Rochester, New York

[8] University Teaching Hospitals Neurology Research Office, Lusaka, Zambia

*Correspondence

maria.bernabeu@embl.es

viola.introini@embl.es

Extended Data

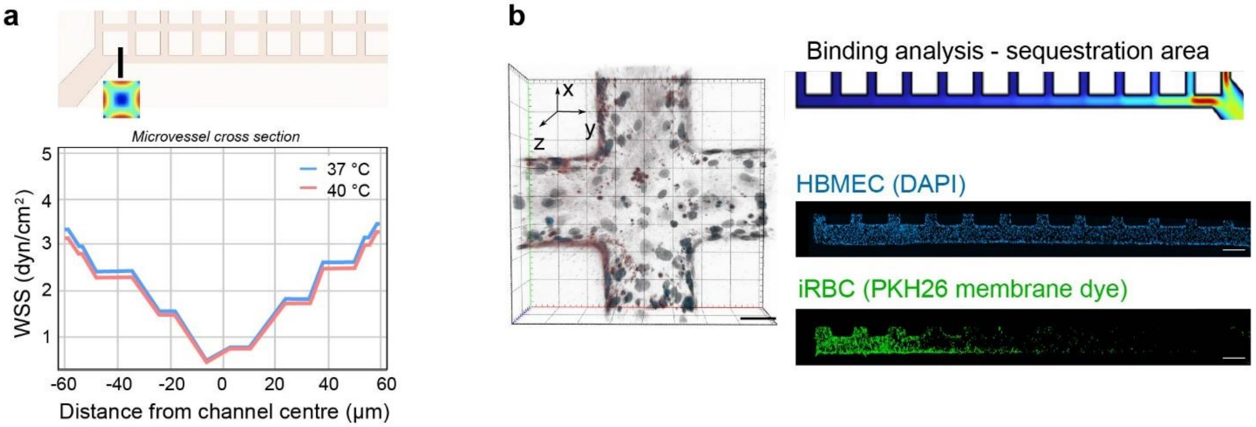

Figure S1. Wall shear stress (WSS) profile and binding of uninfected RBCs in microvessels. a, Lumen cross-sectional WSS profile across the first branch of the microvessel network (black line), simulated based on medium viscosity and density at either 37 °C or 40 °C using COMSOL software prior to seeding of endothelial cells (see Methods). **b,** Reconstruction of z-stack immunofluorescence image labelling with anti-glycophorin A antibody (red) for iRBCs and nuclear staining by DAPI (blue). Binding analysis of iRBC sequestered area along the grid edges (green). Endothelial coverage is confirmed by DAPI labelling.

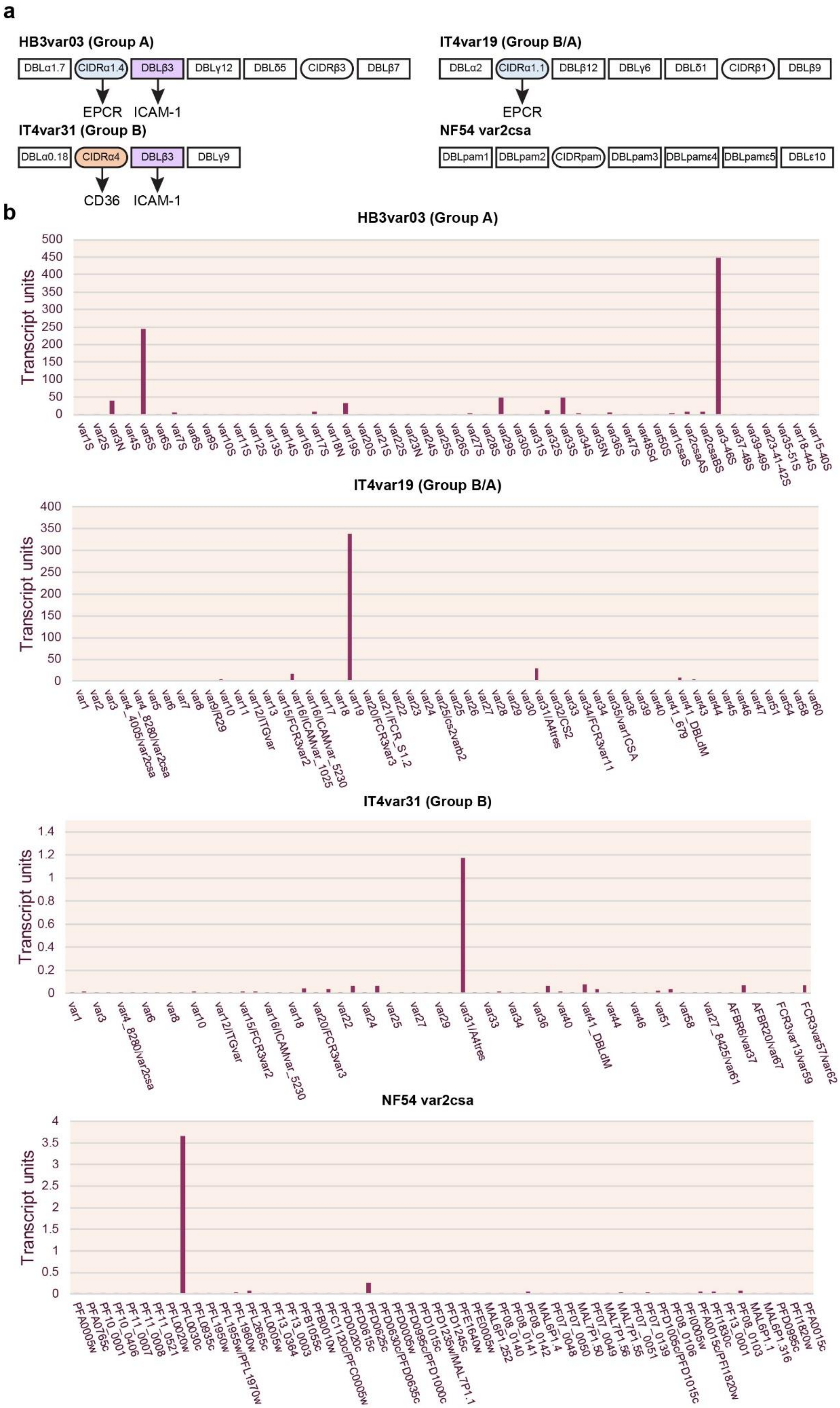
a
HB3var03 (Group A)
DBLα1.7
CIDRα1.4
DBLβ3
DBLγ12
DBLδ5
CIDRβ3
DBLβ7
EPCR
ICAM-1
IT4var19 (Group B/A)
DBLα2
CIDRα1.1
DBLβ12
DBLγ6
DBLδ1
CIDRβ1
DBLβ9
EPCR
IT4var31 (Group B)
DBLα0.18
CIDRα4
DBLβ3
DBLγ9
CD36
ICAM-1
NF54 var2csa
DBLpam1
DBLpam2
CIDRpam
DBLpam3
DBLpamε4
DBLpamε5
DBLε10
b
HB3var03 (Group A)
Transcript units
IT4var19 (Group B/A)
Transcript units
IT4var31 (Group B)
Transcript units
NF54 var2csa
Transcript units

Figure S2. *var* architecture and transcription profile of *P. falciparum* lines. a, Schematic representation of the PfEMP1 preferentially expressed by each experimental *P. falciparum* parasite line. Domain binding activity to endothelial receptors are indicated by arrows. **b,** The var gene transcription profile of ring-stage iRBCs was analysed by qRT-PCR with IT4[1], HB3[2], and 3D7[3] (NF54 strain is identical to the NF54-derived clone 3D7) *var* strain-specific primer sets, as previously published[4,5]. Transcription unit levels are normalised to the housekeeping control gene STS (seryl-tRNA synthetase).

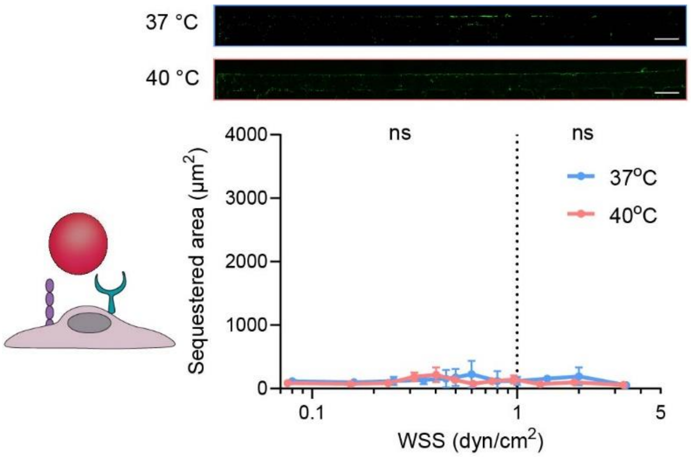

Figure S3. Uninfected RBC binding to HBMEC microvessels at 37 and 40 °C across physiological WSS. Medians are represented by dots and interquartile range by error bars. Statistical analysis of binned regions (< 1 dyn/cm^2 and ≥ 1 dyn/cm^2) (dotted line) was determined by Mann-Whitney U test (n = 4 biological replicates). Scale bars = 200 µm.

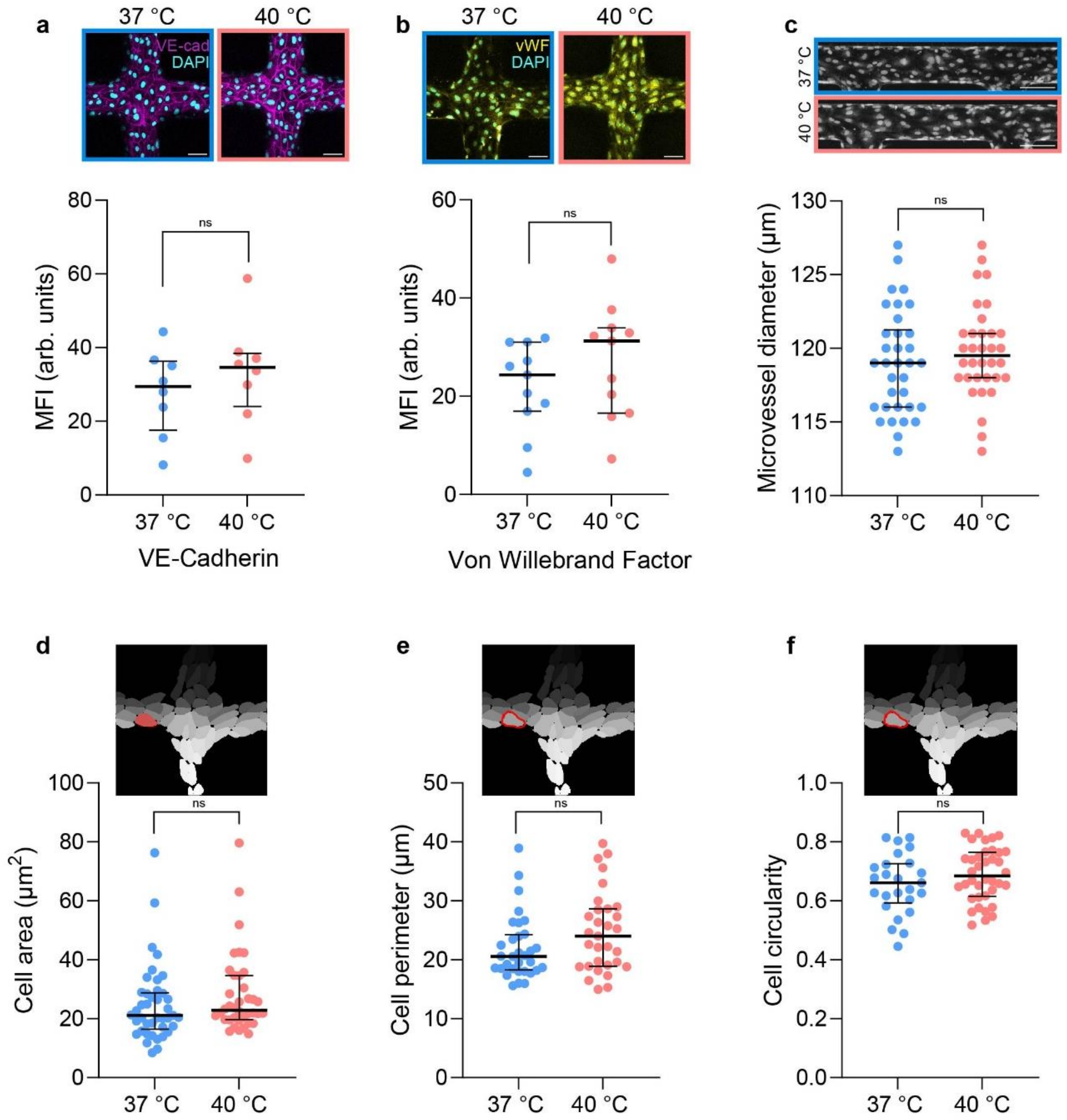

Figure S4. Characterisation of the effect of febrile temperature on endothelial junctions, von Willebrand Factor release and microvessel morphology. a, Z-projection of immunofluorescence images of 3D microvessels incubated for 1 hour at either 37 °C or 40 °C showing VE-cadherin (magenta) or **b,** von Willebrand Factor (vWF, yellow) and DAPI staining (cyan) (top). Scale bars = 50 µm. Corresponding mean fluorescence intensity (MFI) of VE-cadherin or vWF at either 37 °C or 40 °C where each data point represents one device (bottom). **c,** Representative images (top) and quantification of the diameter of 3D microvessels located along grid edges (bottom) at 37 °C or 40 °C. Scale bar = 100 µm. Quantification of HBMEC cell area **(d)**, perimeter **(e)**, and circularity **(f)** in microvessels exposed to 37 °C or 40 °C, with measurements taken from both top and bottom microvessel surfaces of 4 distinct devices. Bars represent

mean ± standard error of the mean (SEM) and statistical significance was assessed by Mann-Whitney U test (**a-f**).

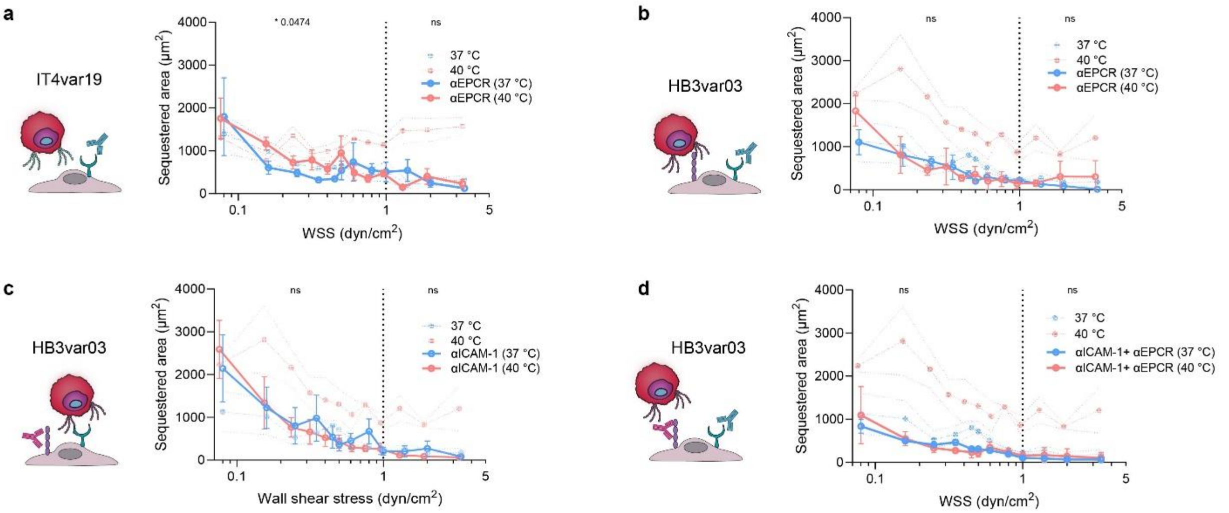

Figure S5. Effect of EPCR and ICAM-1 blockade on *P. falciparum*-iRBC binding under normothermic and febrile conditions. a, Left: cartoon of anti-EPCR antibody inhibition of IT4var19 strain iRBC binding. Right: sequestered area of IT4var19 iRBCs at 37 °C and 40 °C across WSS, with or without microvessel treatment using an anti-EPCR mAb 252. **b,** Left: cartoon of anti-EPCR antibody inhibition of HB3var03 strain iRBC binding. Right: Sequestered area of HB3var03 iRBCs at 37 °C and 40 °C across WSS, with or without microvessel treatment w ;ith anti-EPCR mAb 252. **c,** Left: cartoon of anti-ICAM-1 antibody inhibition of HB3var03 strain iRBC binding. Right: Sequestered area of HB3var03 iRBCs at 37 °C and 40 °C across WSS, with or without microvessel treatment with anti-ICAM-1 mAb 15.2. **d**, Left: cartoon of anti-ICAM-1 and anti-EPCR antibodie inhibition of HB3var03 strain iRBC binding. Right: Sequestered area of HB3var03 iRBCs at 37 °C and 40 °C across WSS, with or without microvessel treatment with both antibodies. Medians and interquartile ranges are shown. Only statistical analysis between antibody treated-microvessels at 37 °C and 40 °C is reported for WSS binned regions < 1 and ≥ 1 dyn/cm^2, and determined by Mann-Whitney U test (n = 4-6 independent biological replicates per condition).

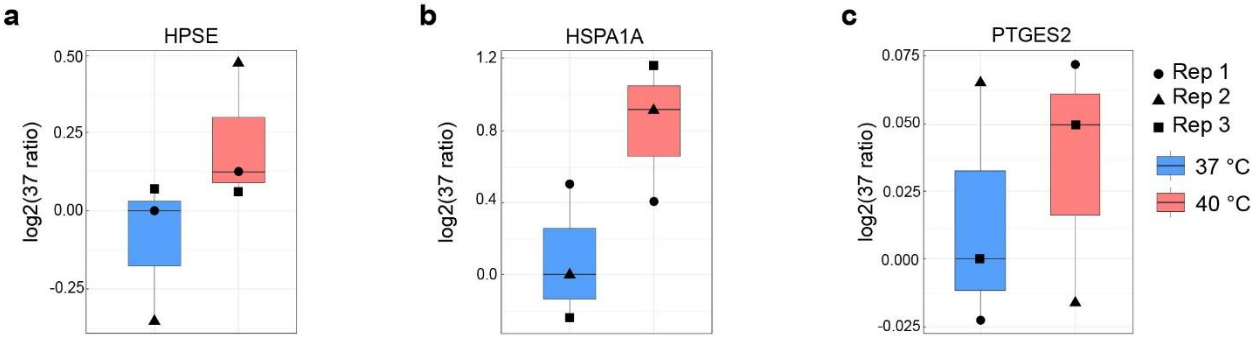

Figure S6. Proteomic alterations in HBMEC exposed to 37 °C or 40 °C for 1 h, highlighting proteins linked to febrile stress and endothelial glycocalyx shedding. **a**, Heparanase (HPSE); b, Heat shock protein HSP70-1A (HSPA1A); **c**, Prostaglandin E synthase 2 (PTGES2). Shown are the ratio of the normalised TMT reporter ion intensity relative to the median of the 37 °C value. *n* = 3 independent biological replicates.

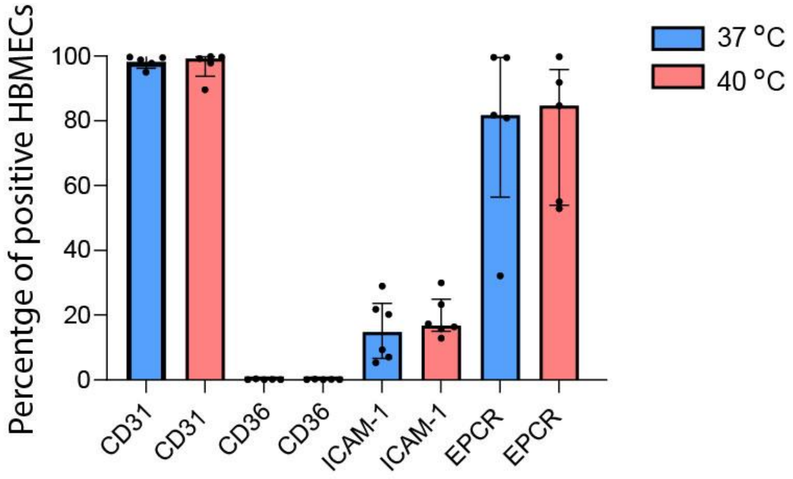

Figure S7. HBMEC surface expression of endothelial receptors involved in iRBC binding. Percentage of HBMECs expressing surface markers by flow cytometry after a 1h incubation at 37 or 40 °C. Bars represent mean ± standard deviation (SD) (n = 5-6 biological replicates).

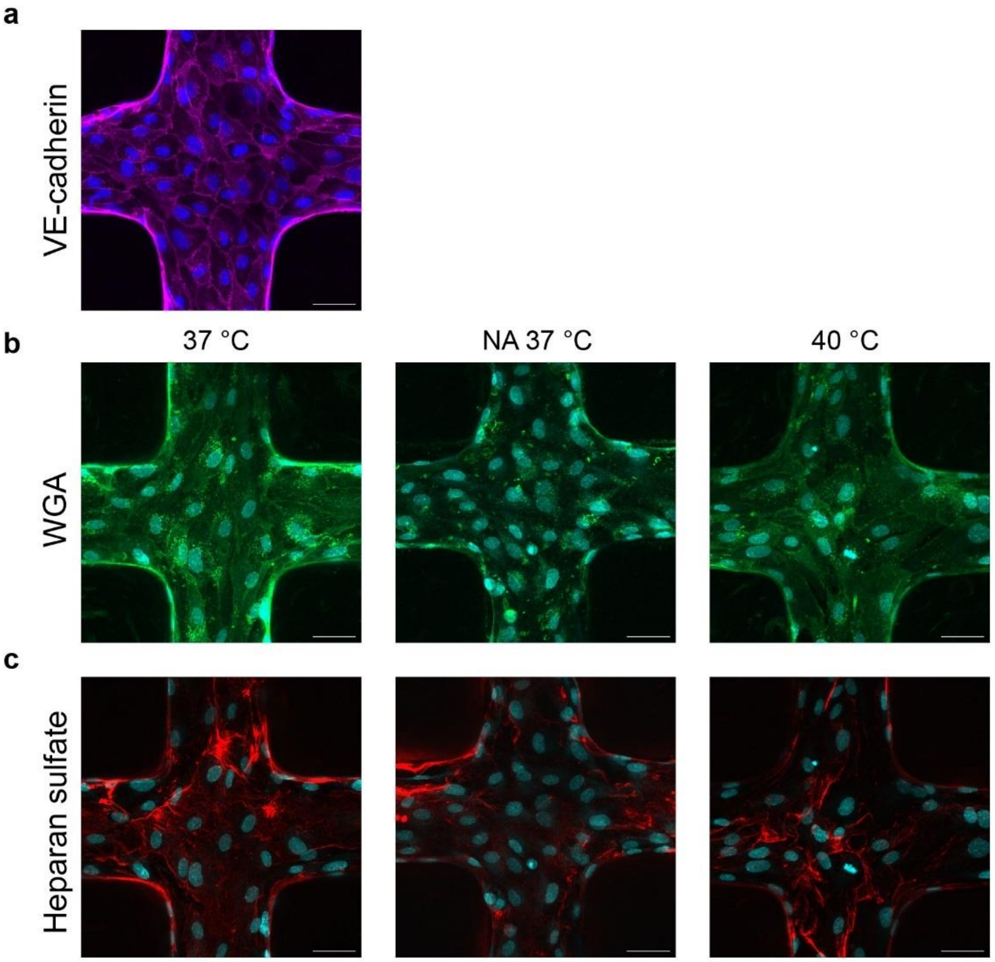

Figure S8. Glycocalyx of 3D lung microvessels. Representative z-projection immunofluorescence images of 3D lung microvessels stained for **a,** junctional marker VE-Cadherin, and glycocalyx components **b**, sialic acid (WGA) and **c,** heparan sulfate and nuclei (DAPI, cyan). Images b-c following incubation at 37 °C, 40 °C for 1 h, or neuraminidase (NA) treatment for 30 min at 37 °C. Scale bars = 50 µm.

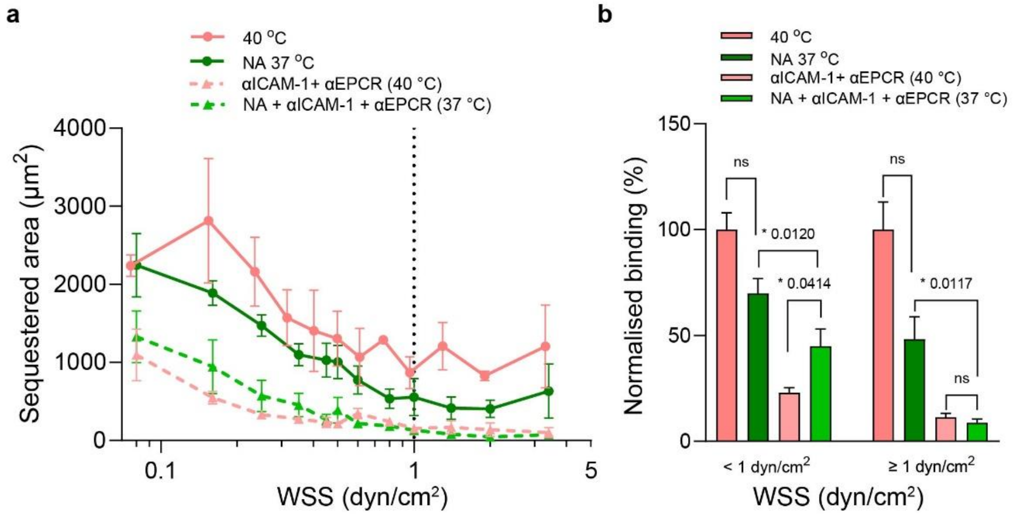

Figure S9. Effect of EPCR and ICAM-1 blockade on *P. falciparum*-iRBC binding following NA-mediated disruption of the glycocalyx. a, Binding of HB3var03 strain iRBCs at 40 °C or 37 °C following microvessel NA treatment ± anti-ICAM-1 mAb 15.2 and anti-EPCR mAb 252 antibodies. Medians are represented by dots and interquartile range by error bars. **b,** Percentage of binding of HB3var03 iRBCs at 40 °C or following NA treatment at 37 °C (filled) and upon anti-ICAM-1 mAb 15.2 and anti-EPCR mAb 252 antibodies (stripes). Bars represent mean ± SEM. Statistical analysis of binding within each WSS binned region (≥ or < 1 dyn/cm^2) was determined by Mann-Whitney U test.

References

1. Janes JH, Wang CP, Levin-Edens E, et al. Investigating the Host Binding Signature on the Plasmodium falciparum PfEMP1 Protein Family. *PLOS Pathogens*. 2011;7(5):e1002032. doi:10.1371/journal.ppat.1002032

2. Soerli J, Barfod L, Lavstsen T, Bernasconi NL, Lanzavecchia A, Hviid L. Human monoclonal IgG selection of Plasmodium falciparum for the expression of placental malaria-specific variant surface antigens. *Parasite Immunol*. 2009;31(6):341-346. doi:10.1111/j.1365-3024.2009.01097.x

3. Rask TS, Hansen DA, Theander TG, Pedersen AG, Lavstsen T. Plasmodium falciparum Erythrocyte Membrane Protein 1 Diversity in Seven Genomes – Divide and Conquer. *PLOS Computational Biology*. 2010;6(9):e1000933. doi:10.1371/journal.pcbi.1000933

4. Gillrie MR, Renaux B, Russell-Goldman E, et al. Thrombin Cleavage of Plasmodium falciparum Erythrocyte Membrane Protein 1 Inhibits Cytoadherence. *mBio*. 2016;7(5):e01120-16. doi:10.1128/mBio.01120-16

5. Bernabeu M, Gunnarsson C, Vishnyakova M, et al. Binding Heterogeneity of Plasmodium falciparum to Engineered 3D Brain Microvessels Is Mediated by EPCR and ICAM-1. *mBio*. 2019;10(3):e00420-19. doi:10.1128/mBio.00420-19
